# A Look inside a Flexible Open-Source Scanning Electrochemical Probe Microscope

**DOI:** 10.1021/acselectrochem.5c00354

**Published:** 2025-12-04

**Authors:** Kim McKelvey, Martin Andrew Edwards, Minkyung Kang, Marc Brunet Cabré, Nicholas B. Jones, Patrick R. Unwin

**Affiliations:** † MacDiarmid Institute for Advanced Materials and Nanotechnology, School of Chemical and Physical Sciences, 8491 Victoria University of Wellington, Wellington 6012, New Zealand; ‡ Department of Chemistry and Biochemistry, 3341 University of Arkansas, Fayetteville, Arkansas 72701, United States; § Department of Chemistry, 2707 University of Warwick, Coventry CV4 7AL, United Kingdom; ∥ School of Chemistry, 4334 University of Sydney, Camperdown, NSW 2006, Australia; ⊥ School of Chemistry, Trinity College Dublin, Dublin 2, Ireland

**Keywords:** Scanning electrochemical cell microscopy, scanning electrochemical microscopy, scanning ion conductance microscopy, instrumentation, nanoelectrochemistry, electrochemical imaging

## Abstract

Scanning electrochemical probe microscopy (SEPM) maps and investigates the chemical and physical properties of surfaces and interfaces using a micro- or nanoscale electrochemical probe, e.g., a microelectrode or a nanopipette, positioned close to an interface of interest. SEPM instruments share a common general architecture but are distinct from each other through the choice of probe and in the different physicochemical properties of the sample that can be investigated, including, among others, interfacial charge transfer rates, topography, permeability, or surface charge. Thus, a single instrument, with an appropriately flexible control system, can facilitate widespread access to the family of SEPM techniques—scanning electrochemical microscopy (SECM), scanning ion conductance microscopy (SICM), scanning electrochemical cell microscopy (SECCM), the scanning micropipette contact method (SMCM), and hybrid varieties of these techniques. Herein, we describe in detail a *flexible open-source SEPM instrument* that can perform *common and widely applicable SEPM techniques and experimenter-defined methodologies, with minimal programming from the user.* The instrument makes use of a field programmable gate array (FPGA)-based data acquisition card, and this contribution further illustrates the benefits of adopting FPGA architecture generally in electrochemical instrumentation. We describe the software and hardware for the instrument, using examples from the literature to illustrate how common SEPM operation modes and hyphenated techniques are readily implemented. Additionally, to demonstrate the application of custom-developed scanning protocols, we briefly present some further experimental examples. This Tutorial seeks to serve the needs of expert users of SEPMs and encourage new entrants alike. To this end, to encourage those who are interested in either setting up their own instruments or making optimal use of commercially available instruments, we also briefly include some more basic and general information on SEPM techniques and uses, to put the more advanced work and instrumental aspects in context.

## Graphical abstract

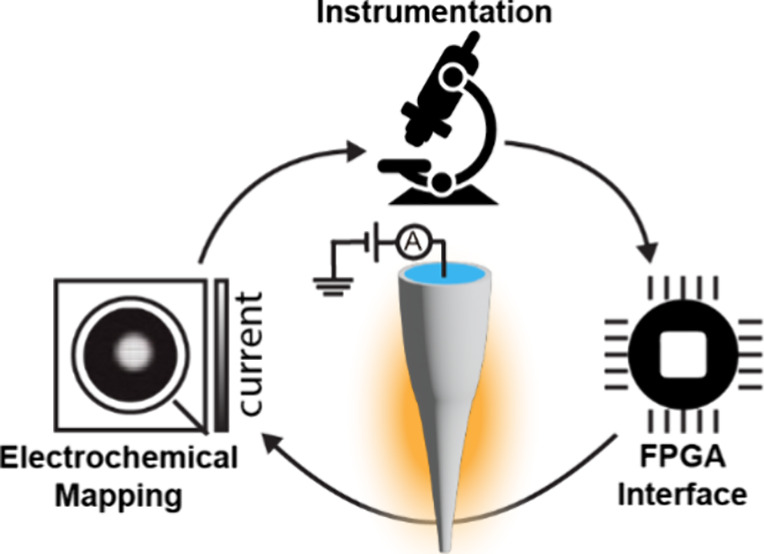

## Introduction

Scanning electrochemical probe microscopy (SEPM) is a family of closely related scanning probe microscopy (SPM) techniques.^1^ In SEPM, micro- or nanometric probes containing one or more electrodes are scanned in close proximity to an interface of interest. Electrochemical signals (either current or potential) measured at the electrodes are recorded as a function of the probe position to map physicochemical properties of the sample. A wide variety of properties, many of which are inaccessible to other forms of microscopies, e.g., electrode activity, chemical flux, permeability, and surface charge, can be visualized *in situ* through judicious application of SEPM and analysis of the resultant data.^2–6^ In addition to mapping the properties of a sample, the probe can be used to evaluate the lifetime of surface-generated species^7^ and to manipulate or modify the sample, e.g., by electrogeneration of an etchant,^8,9^ or through electrodeposition.^10,11^

The SEPM family of techniques comprises scanning electrochemical microscopy (SECM),^3^ scanning ion conductance microscopy (SICM),^2^ scanning electrochemical cell microscopy (SECCM),^12^ and the scanning micropipette contact method (SMCM).^13,14^ There are also hybrid versions of these techniques—with each other (as considered herein)^4^—and with other SPMs, such as atomic force microscopy (AFM).^15,16^ SEPMs have a common general instrument anatomy (*vide infra*), differing only in the type of probe used and the mode of operation (movement strategy, applied potential waveform, feedback signal, etc.). Over the past decades, many different modes of operation for measuring different physicochemical properties have been introduced.^2–6^ Typically, a research group will build and/or program a home-built instrument to perform a new or custom technique, or will purchase a commercial electrochemical instruments, such as those from CH Instruments, Metrohm, Princeton Applied Research, Sensolytics, HEKA, and Park Systems. The latter provide reliable and standardized platforms for scanning electrochemical measurements but restrict users to the hardware platform and techniques provided by the supplier. This limits the choice of techniques available to an experimenter and will also mean many experimental groups only have historic techniques available to them. Crucially, this limits the rate at which new methodologies can be developed and adapted.

To address these challenges, we developed a flexible SEPM instrument capable of performing both common SEPM techniques and experimenter-defined methodologies, all with minimal programming. The platform has been adopted and used successfully by an increasing number of groups,^17,18^ and SEPMs are more generally undergoing a resurgence of interest driven by new methods and capabilities, and applications in materials science, energy science^19,20^ and the life sciences.^21–23^ These applications are underpinned by a desire to understand electrochemical and interfacial processes at the nanoscale or single-entity level,^24–27^ on the one hand, and to enable high-throughput electrochemical analysis on the other.

Herein, we describe the open-source instrument, which comprises: (i) control software, which is offered open-source (academic use license)^28^ to the scientific community as the Warwick Electrochemical SPM (WEC-SPM); and (ii) typical hardware components. The design of the instrument allows easy interchange of hardware components, and where fabrication of custom items is required, editable open-source 3D designs and modes of fabrication are provided. Using experimental examples, we highlight the ease with which this instrument can perform both widely used and customized SEPM techniques. To demonstrate the full repertoire of the instrument, we include two example applications of custom movement and potential schemes in SICM, which enable *in situ* topography and ion flux mapping.

WEC-SPM complements the excellent open-source scanning probe microscopy frameworks previously reported, including a digital signal processor for SPM,^29^ an open-source AFM head,^30^ and data acquisition and visualization systems, such as GXSM.^31^ Compared to these systems, WEC-SPM focuses exclusively on SEPM approaches, with fast, high-precision probe control and data collection. Previously reported low-cost open-source SECM implementations^32,33^ present an even more accessible entry point into SEPM, but lack the same flexibility and high-performance attributes of the instrument described herein.

## Background to SEPM

### The SEPM Family

Following the invention of scanning tunneling microscopy (STM)^34^ as the first SPM, there was a flurry of activity in which a variety of different probe-based microscopy methods were reported in the following years, including the use of scanned electrode (electrochemical) probes.^3,6,35^ An early example in the field of electrochemistry consisted of a micropipette filled with a solution containing a redox-active species, scanned above an electrode bathed in a solution lacking the redox species.^36,37^ Subsequently, Engstrom used a mobile microelectrode as a scanned probe to map the diffusion layer of a larger electrode.^38^ This pioneering work led Bard and co-workers to invent scanning electrochemical microscopy (SECM), shown schematically in Figure 1A.^35,39,40^ In typical SECM measurements, an inlaid disk microelectrode is scanned above a substrate bathed in electrolyte while measuring a faradaic current, which can be used to map quantities such as local concentration,^41^ electron-transfer rates,^42,43^ rates of (bio)­chemical reactions,^21^ and more.^3,6^ Although many applications of SECM operate under steady-state conditions,^3,35^ transient techniques (e.g., potential step chronoamperometry) can be implemented to facilitate surface titrations. Transient techniques were first applied to studies of adsorption/desorption kinetics and surface diffusion methods^44^ and then extended to the surface (redox) interrogation of surface chemistry, lateral charge transfer, and the detection of bound intermediates.^45–47^

**Figure 1:**
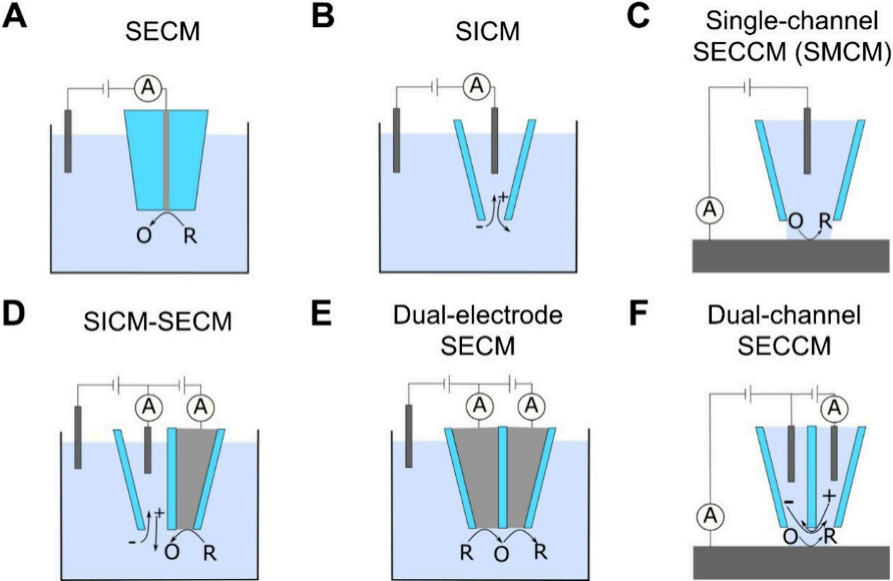
Representation of the probes used in common SEPMs, indicating the electron transfer (R ⇋ O + n*e* ^–^) or ion migration current (cation: + and anion: – migration indicated by arrows) that dictates the electrochemical response. Key: grey - electrode, cyan - glass, light blue - electrolyte, white - air/gas. Note that C and F examples do not require immersion of the probe and entire electrode surface in electrolyte, while the others work in the solution phase. Abbreviations: SECM - scanning electrochemical microscopy, SICM - scanning ion conductance microscopy, SECCM - scanning electrochemical cell microscopy, SMCM - scanning micropipette contact method. Note that single-channel (single-barrel) SECCM was originally called SMCM but is now mainly referred to as SECCM, and so we adopt this terminology. The technique shown in part F is sometimes referred to as dual-channel (dual-barrel) SECCM to distinguish itself from the technique shown in part C. It should further be noted that dual-channel SECCM can be used to study interfacial ion transfer reactions.

Scanning ion conductance microscopy (SICM), introduced by Hansma and co-workers,^48,49^ uses an electrolyte-filled nanopipette to map sample topography, as is shown in Figure 1B. The ionic current between two electrodes, one inside the pipette, another outside, provides an electrical signal which (typically) decreases as the pipette is approached to a surface.^2^ Further developments^50^ have used non-constant applied potentials (e.g., bias modulation^51^), which extended SICM to provide complementary physio-chemical information, such as surface charge mapping^52^ and reaction rate mapping.^53^

Most recently, extending traditional scanning capillary techniques used in corrosion science,^54,55^ we introduced the non-immersive scanning micropipette contact method (SMCM), primarily now known as single-channel SECCM,^14^ and dual-channel SECCM.^12,56^ In these techniques, a single- or double-barrel nanopipette filled with electrolyte solution makes meniscus contact with a substrate (usually, but not exclusively, an electrode) surface, to define the area of the substrate that is electrochemically probed, as shown in Figure 1C and F. The nanopipette size, as characterized by the end radius, varies from ∼10 nm to ∼1 μm. This radius defines the meniscus size and thus the spatial resolution of SECCM on many different surfaces.^5,57^ Under certain conditions (such as high pH in aqueous solution), more extensive wetting of the surface may occur and can be characterized.^58^ SECCM avoids bathing the entire sample in solution, which ensures a new region of the surface is accessed for each measurement, enabling the initial steps of solution-induced processes to be studied.^59–63^

In addition to the family of techniques described above, there is a rich tradition of combining individual SEPMs to make hyphenated techniques. Multi-functional probes are fabricated to contain additional pipette barrels and/or electrodes (e.g., SICM-SECM^64–66^ (Figure 1D), dual-electrode SECM^67,68^ (Figure 1E), or SECM-SECCM).^69–71^ Alternatively, a scanned probe is combined with another physicochemical analysis (e.g., mass spectrometry,^72–74^ SICM-SNOM (scanning near-field optical microscopy),^75^ or optical microscopy^76–78^ ), which together allow additional complementary channels of information to be recorded. One of the earliest such combinations was SECM and AFM to make an instrument with AFM tips containing integrated electrodes, which could simultaneously record AFM-related signals and SECM signals.^15,16,79^ Since this technique requires an AFM, for which the architecture is different in some respects from that of SEPMs, we discuss AFM-SECM only briefly in this article, and point the interested reader to a general review on multifunctional AFM.^80^

## Operation of an SEPM: Movement Schemes

To form an SEPM image, the probe (which is also called the tip) is placed in close proximity to the substrate and a quantity, typically the current (sometimes as a function of potential/time), is recorded point-by-point over an array of *x*-*y* positions (where *x* and *y* are orthogonal coordinates in the plane of the sample). Control of the *z*-position (position normal to the substrate) as the probe is moved from point to point, can employ one of several strategies; the most common variants are shown in Figure 2.

**Figure 2:**
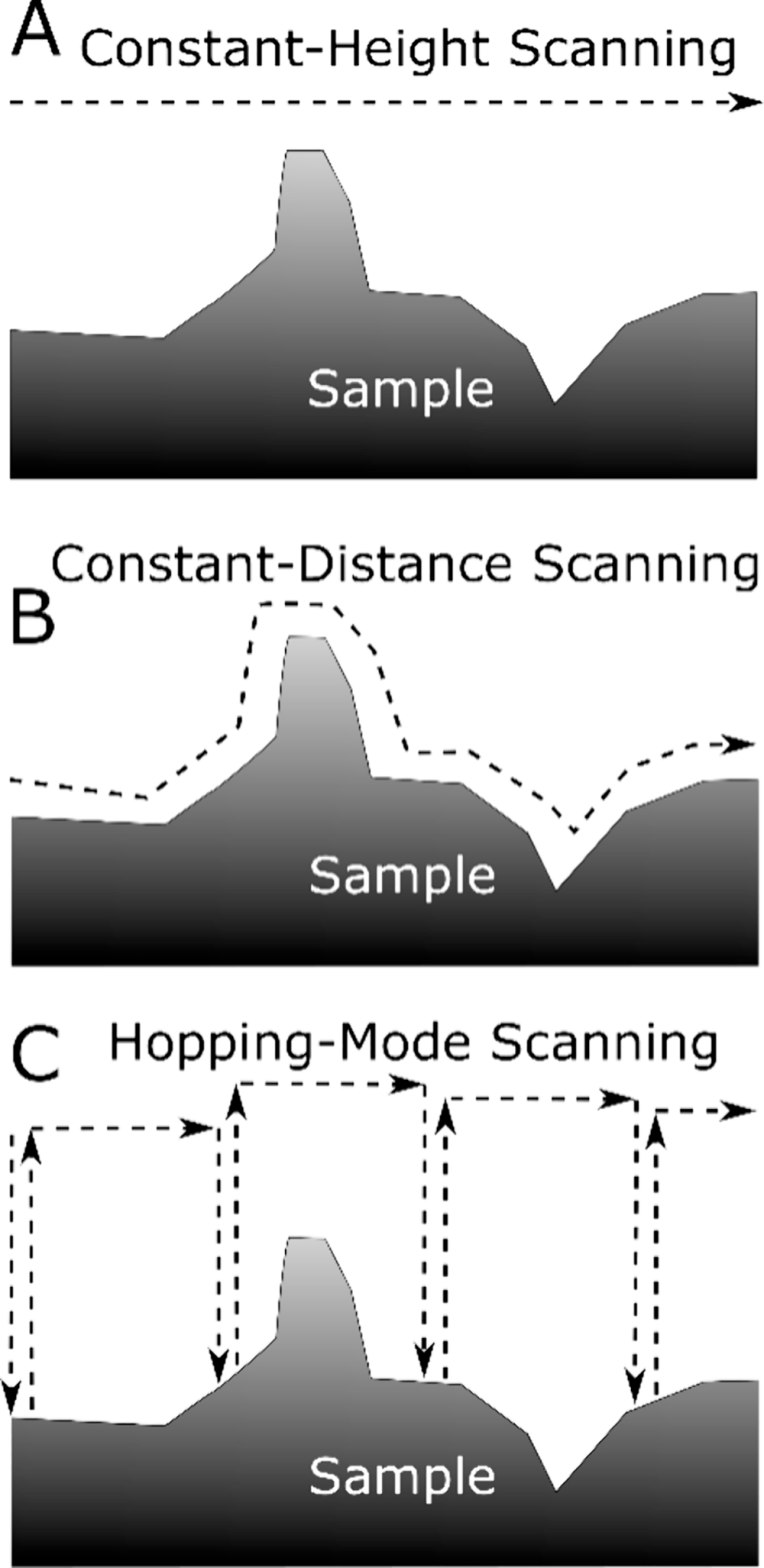
Common probe movement protocols used in scanned probe microscopy, all of which are available within the software. The movement of the tip of the probe is indicated by the dashed lines.

Early SECM measurements used a constant-height mode (Figure 2A), in which the *z*-position is fixed by the experimenter with the sample or tip scanned in the *x–y* plane.^35^ This remains the main SECM imaging mode. While its simplicity makes it easy to implement experimentally, it has several drawbacks. If a sample is rough or tilted, the probe may collide with the sample surface, causing damage to one or both. This is especially problematic with small probes, which must be placed very close to the surface (typically within a distance that is of the size of, or smaller than, the radius of the active part of the tip) for optimal resolution.^81,82^ Moreover, data from constant-height SECM is challenging to interpret, as the current measured can vary due to the tip-sample separation, a change in sample activity, or both. This is the so-called ‘activity-topography convolution’.

Several creative methods were developed to independently determine the tip-sample separation in SECM,^15,64,66,78,83–90^ in an attempt to allow the reliable scanning of a small probe close to an arbitrary interface. These methods required the inclusion of a feedback loop, in which a particular signal (or quantity) is continuously monitored that is a function of the probe-sample separation, while continually adjusting the *z*-position to keep this quantity constant (at a particular value or ‘set point’, *vide supra*). When functioning correctly, the feedback loop maintains a constant tip-sample separation (Figure 2B). This mode is sometimes referred to as constant-distance imaging, with the *z*-position vs *x–y* referred to as the topographical image. Feedback quantities may be electrochemical or non-electrochemical. The ionic current was used as a feedback parameter in the earliest versions of SICM,^48^ while feedback based upon an additional (modulating) electrochemical signal,^83–85,90^ shear force,^78,86^ and (intermittent)^89^ contact^15,87,88^ between the probe and the substrate was also used in SECM imaging.

The hopping-mode of positioning a probe (*vide supra*), shown schematically in Figure 2C was simultaneously developed in 2009 for SICM and SECCM imaging applications.^14,91^ In SECCM, hopping mode was first implemented in ref 14, although not explicitly named as such, while in the same year it was clearly described and named for SICM in ref 91. This approach was introduced to overcome problems associated with imaging very rough samples^91^ and measuring using a discontinuous feedback signal,^14,92^ respectively. In hopping mode, the probe is approached vertically to the surface until a threshold value is passed (e.g., *i* > *i*_threshold_), indicating a successful approach of the probe to the sample surface; an electrochemical characterization may then be performed. The probe is then retracted vertically, moved laterally to a new *x–y* coordinate, and the cycle repeated.^14,91,93,94^

For completeness, it should be mentioned that, Korchev, Klenerman, and co-workers reported a robust SICM operating mode, in which the electrical feedback signal is an oscillating current induced by the oscillation of the pipette in the direction normal to the sample, and detected by lock-in amplification.^95,96^ A mode similar to this was used in the early version of dual-channel SECCM.^12,56,61^

## The Anatomy of an SEPM

In its most basic form, an SEPM consists of an electrochemical probe (e.g., a micro/nano-electrode or -pipette), a 3D positioning system, a current/voltage amplifier, a controller that commands the positioners and applied potential/current, and a method for recording the data (typically integrated into the controller). Figure 3A shows a flexible SEPM instrument with these key components and some common optional components.

**Figure 3:**
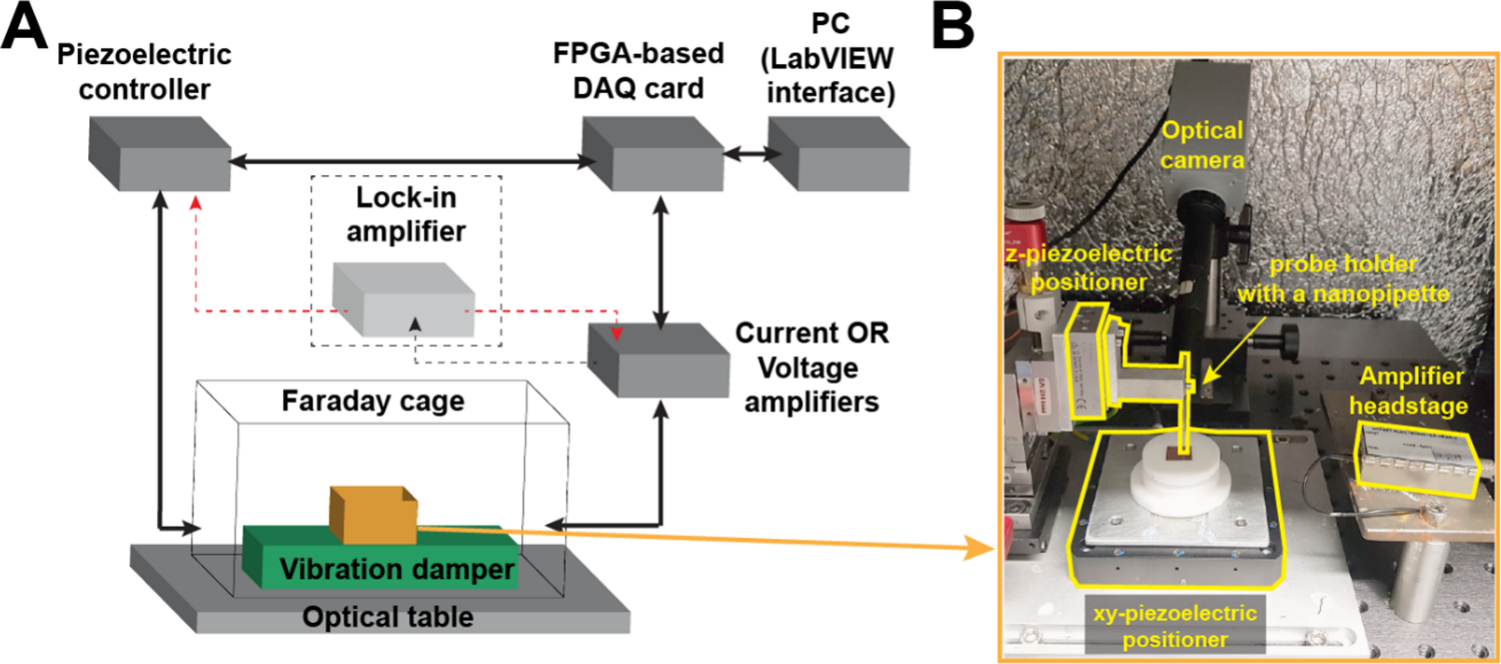
(A) Schematics of an SEPM instrument, showing the major components of the instrument. Note: the lock-in amplifier is optional for distance/voltage modulation feedback mode. (B) Photograph of an SEPM setup in a Faraday cage.

The conserved architecture encouraged us to design a single instrument capable of performing many SEPM techniques. We have made this available to the research community via an academic-use open-source license agreement^28^ to improve accessibility to SEPMs and encourage innovation in the field. Below, we describe the individual physical and software components of the instrument and how they are connected together.

The instrument uses analog voltages to communicate between components, allowing the active components to be chosen to suit a particular experiment without restriction to a single manufacturer (see Supporting Information, section S1 for suggested suppliers, part numbers, etc.). The instrument can perform all the common SEPM experiments.

### 3D Positioning System

3D control of the relative position of the probe with respect to the sample is typically achieved using a combination of fine and coarse positioning. Coarse positioning (∼10 μm to ∼1 cm), which is typically only used between experiments, is achieved using 3-axis linear translation stages equipped with micrometers or finely threaded adjustment screws (either manually or electronically controlled). Fine positioning of the probe with respect to the sample (∼0.1 nm to ∼100 μm), as performed during imaging experiments, uses piezoelectric positioners with an accompanying amplifier.

In the instrument shown in Figure 3B, the lateral and vertical movements are separated, with the sample mounted on XY piezoelectric positioners, while the SEPM tip is mounted on a vertical piezoelectric positioner above the sample surface, which together provide 3D control of the separation between the tip and substrate. Alternatively, all three axes may be controlled by a single piezo unit, which may move either the tip or the sample.

### Current/Voltage Amplifier

A current/voltage amplifier is used to measure and amplify the electrochemical signal(s) of interest. Homebuilt or commercial amplifiers or potentiostats/galvanostats can be used, with various sensitivity scales typically ranging from pA to nA (current) or μV to V (potential). The quantity measured by the amplifier is passed to the controller (e.g., field-programmable gate array data acquisition card (FPGA-based DAQ card)) as a voltage, where appropriate scaling parameters are used to determine the physical value. The potential/current is applied to the working electrode through the amplifiers or directly to the reference electrode (depending on the electronic circuit configurations) via an analog signal from the controller. NB: In many SEPM experiments, the electrochemical current is sufficiently low (typically ≪ 10 nA) that a two-electrode setup of a working electrode and a quasi-reference/counter electrode (QRCE)^97^ is used.

### Instrument Control and Data Acquisition

A general flexible SEPM requires control hardware that can measure, record and react to the electrochemical response. To communicate with the instrumentation (piezo positioners and current/voltage amplifiers, plus any auxiliary components), which can come from any of a variety of manufacturers, analog voltages (−10 to +10 V) are used to command and receive information from the devices. These voltages are converted through linear scaling to represent physical quantities (amperes, volts, nanometers, etc.). In this way, the control unit is agnostic to the origin or destination of a signal, facilitating flexibility in all components.

An FPGA-based DAQ card attached to a computer running LabVIEW is used to generate control signals and record amplified electrochemical signals.^98,99^ This card contains reconfigurable hardware that can run a custom program and acquire/output voltages. Separating these components from the central processing unit of the computer, which might be running other software, allows a fast, stable feedback response, and the flexibility to implement many different feedback schemes (the PCIe-7852R and USB-7855R FPGA-based DAQ cards used in the instrument run with an 80 MHz clock cycle and deliver multiplexed 750 kS/s or 1 MS/s analog signals, respectively). A detailed scheme of all electrical connections of the system is listed in the Supporting Information, S3.

### Sample and Probe Holders

The probe (see *The SEPM Family* section) and sample must both be securely mounted within the SEPM so that the only movement during imaging is from the 3D-positioning system. Probe/sample holders are typically custom-made, depending on the probe type and the sample being used. Editable open-source designs for a probe holder and other components requiring custom fabrication are described in the Supporting Information, S2. If appropriate expertise and machinery are available, holders can be fabricated in-house. Alternatively, machining-on-demand services allow one to order these components.

### Vibration, Electrical and Thermal Isolation

Without mitigation, external influences such as electromagnetic (EM) radiation or physical vibrations can adversely affect the operation of SEPMs. A Faraday cage (custom fabricated or from a commercial supplier) surrounds the probe and amplifier (and sometimes other components), shielding against EM radiation, which could otherwise induce electric currents in wires that overwhelm the small electrochemical currents.^100^ Extraneous vibrations can cause unintended variation in the tip-sample separation, which can lead to damage through uncontrolled tip-sample contact. Vibrations can also generate electrical noise through capacitive coupling between wires and other electrical components. The SEPM can be isolated from vibrations by mounting it on an active or passive damping table and by lining the Faraday cage with sound-absorbing material (e.g., acoustic foam). Thermal isolation can be achieved by including heat sinks within the cage.^101^

### Lock-in Amplifier and Voltage Adder (Both Optional)

Several SEPM techniques require measurement of the response to an oscillating stimulus (e.g., to an oscillation of the vertical position or potential of the probe). Such measurements began with tip-position modulation in SECM^102^ and were extended to intermittent-contact SECM^89^ and SECCM continuous surface scanning.^12^ As mentioned earlier, such measurements are also the basis for distance- ^95,103^ and potential (bias)^51^-modulation SICM. In these applications, and other similar modes, a lock-in amplifier both generates the driving oscillation and extracts oscillations of the same frequency (or multiples thereof) from the electrochemical signal, which is fed into the lock-in amplifier as a voltage. When applications require the addition of the DC offset to the oscillating component, e.g., when the *z*-position is oscillated above its average position in dual-channel SECCM,^12^ a voltage adder is required (see Supporting Information, S1 (viii) for further details).

### Optics for Alignment (Optional)

To aid in initial alignment of the probe over a region of the sample, it is common to use optical assistance. This is achieved either through overhead or orthogonally mounted cameras^104^ (one of which is shown in Figure 3B) or by mounting the entire instrument on an (inverted) optical microscope.^105–107^ When the SEPM technique is SECCM, the latter approach has been called ‘optically targeted electrochemical cell microscopy’^106^ and is particularly powerful for directing the probe to particular features on a surface. The latter configuration allows for the implementation of sophisticated optical microscopy techniques with SEPM, from fluorescence-based methods^105–113^ to interference reflection microscopy^76,114,115^ and also paves the way for intelligent or autonomous scanning of samples, where the probe seeks out and is guided to regions of interest^77^ by *in situ* or *operando* optical microscopy.

## Control Software

To deliver maximum functionality to the widest range of users, an SEPM should satisfy several key criteria:

1) flexible capabilities - the SEPM should be capable of performing both existing and newly conceived protocols/techniques;
2) responsiveness - the SEPM should be able to react to changes in the current or other quantities, e.g., through closed-loop feedback or reacting once a threshold is reached;
3) ease of use - running protocols and implementing new procedures should not require large amounts of time or advanced programming skills.

To achieve this, the control software, which is described in detail below, uses the strategy of:

1) breaking down complex experiments into elemental steps;
2) using analog voltages to communicate between different components (see Instrument Control and Data Acquisition for details);
3) reusing functionality;
4) layered abstraction.

The concepts used in implementing these strategies are described in detail below.

### Waypoints

A key concept in the control software is that each experiment is broken into elemental steps, so-called ‘waypoints’ (e.g., move the probe to point *x*, sweep potential to *E*_1_, etc.), which each have a selection of options (movement speed, sweep rate, whether to use feedback to keep a quantity constant, halt on reaching threshold current, etc.). Conceptually, these can be considered as points in (*x*, *y*, *z*, *E*) space and instructions on how to move between them. Breaking the experiments down in this way means that each functionality is programmed once by an expert user (details below), and can then be reused as part of a sequence of waypoints to build experiments. When these basic steps are linked together, complex experiments can be designed and performed in a short period of time by an experimenter with limited programming experience.

An example of a cyclic voltammogram (scanned from 0.6 to −0.5 V at 10 mV/s; no quiet time) broken into its elemental components is given in Table 1. For the first waypoint, the potential is stepped to the start potential (*E* = 0.6 V), next the potential is swept to *E* = −0.5 V with a velocity (sweep rate) of ν_E_ = 0.01 V/s, finally the potential is swept back to *E* = 0.6 V again with a velocity (sweep rate) of ν_E_ = 0.01 V/s. Throughout this voltammogram, the position of the probe is held at (*x*, *y*, *z*) = (0, 0, 0) μm.

**Table 1:** A Series of Waypoints That Define a CV; The CV Scans from 0.6 to −0.5 V at a Scan Rate of 10 mV/s while throughout the Tip Is Held at a Position of (0, 0, 0) μm^a^

	Position				Velocity				Step/Sweep potential
Waypoint number	*x* (μm)	*y* (μm)	*z* (μm)	Potential, *E* (V)	*v*_*x*_ (μm/s)	*v*_*y*_ (μm/s)	*v*_*z*_ (μm/s)	Sweep rate, *v*_E_ (V/s)	T/F (Step/Sweep)
1	0	0	0	0.6	0	0	0	0	T(step)
2	0	0	0	–0.5	0	0	0	0.01	F(sweep)
3	0	0	0	0.6	0	0	0	0.01	F(sweep)

a For clarity, waypoint options that are not relevant to voltammetry are omitted (see Supporting Information, section S4 for a complete list of waypoint options). NB: In this example, there is no quiet time before the start of the scan.

### Feedback and Moving between Waypoints

In the voltammogram above, the voltage was swept without the requirement of reacting to the measured response. However, in a general experiment, responding to measured quantities and inputs provides important functionality. For example, in constant-distance scanning (see Figure 2B and discussion), continuous feedback is required to adjust the *z*-position while the probe is moved between positions, while in hopping scanning (Figure 2C), the probe’s approach to the surface must be halted when it is in close proximity, lest it collides with the surface. This latter functionality is achieved by halting movement to a waypoint either once a threshold is met, i.e., the current passes above/below a specific value,^14^ or decreases by a certain percentage from that measured at the start of the movement (far from the surface).^65^

The above examples represent just three examples of the type of feedback (Supporting Information, Table S2) that can be selected whenever a waypoint is being executed: 1) voltammetry employs Feedback Type 0, “no feedback”; 2) constant-distance imaging utilizes Feedback Type 4, “Maintain set point using a proportional controller to adjust the *z*-position”; and 3) hopping imaging employs either Feedback Type 2, “Move to next waypoint once the threshold is achieved”, or Feedback Type 8, “measure the average current value at start of movement (typically in bulk solution), and then pause movement based on percentage deviation from this initial measurement”). The complete list of feedback options, currently consisting of 15 types, is listed in the Supporting Information, Table S2, along with their applications and published references. Note that a CV represents a case where no feedback was used; Feedback Type 0 (no feedback, the default setting), but was omitted from Table 1 for clarity.

Together, the feedback options add responsiveness and immense flexibility to the instrument, expanding the range of experiments that can be performed. While many of the feedback modes were designed with electrochemical imaging in mind, they can also be used for other experiments, e.g., using feedback to continually adjust the applied voltage to maintain an applied current, which is precisely the role of a galvanostat.^116^ Such galvanostatic functionality has been used to characterize stochastic phase nucleation on nanoelectrodes; a current pulse is applied while the applied potential is monitored, the current is returned to zero once nucleation occurs, and a threshold voltage is reached.^117^

### Preparing Waypoints

The list of waypoints is prepared in the LabVIEW programming environment, which easily allows one to directly enter a table (array) like Table 1 (interested users are encouraged to look at the block diagram for “cyclic voltammetry.vi” to see this implemented). For a more complicated experiment, such as an imaging scan, the list of waypoints is longer and will depend on the area scanned/points-per-image, but the individual elements are no different. The anatomy of such experiments typically involves repeated procedures, as shown schematically in Figure 4. An effective strategy for preparing waypoints in such an experiment is to use loops, where the basic input relating to the position is updated once per point measured. A detailed example is presented in section S5 of the Supporting Information.

**Figure 4:**
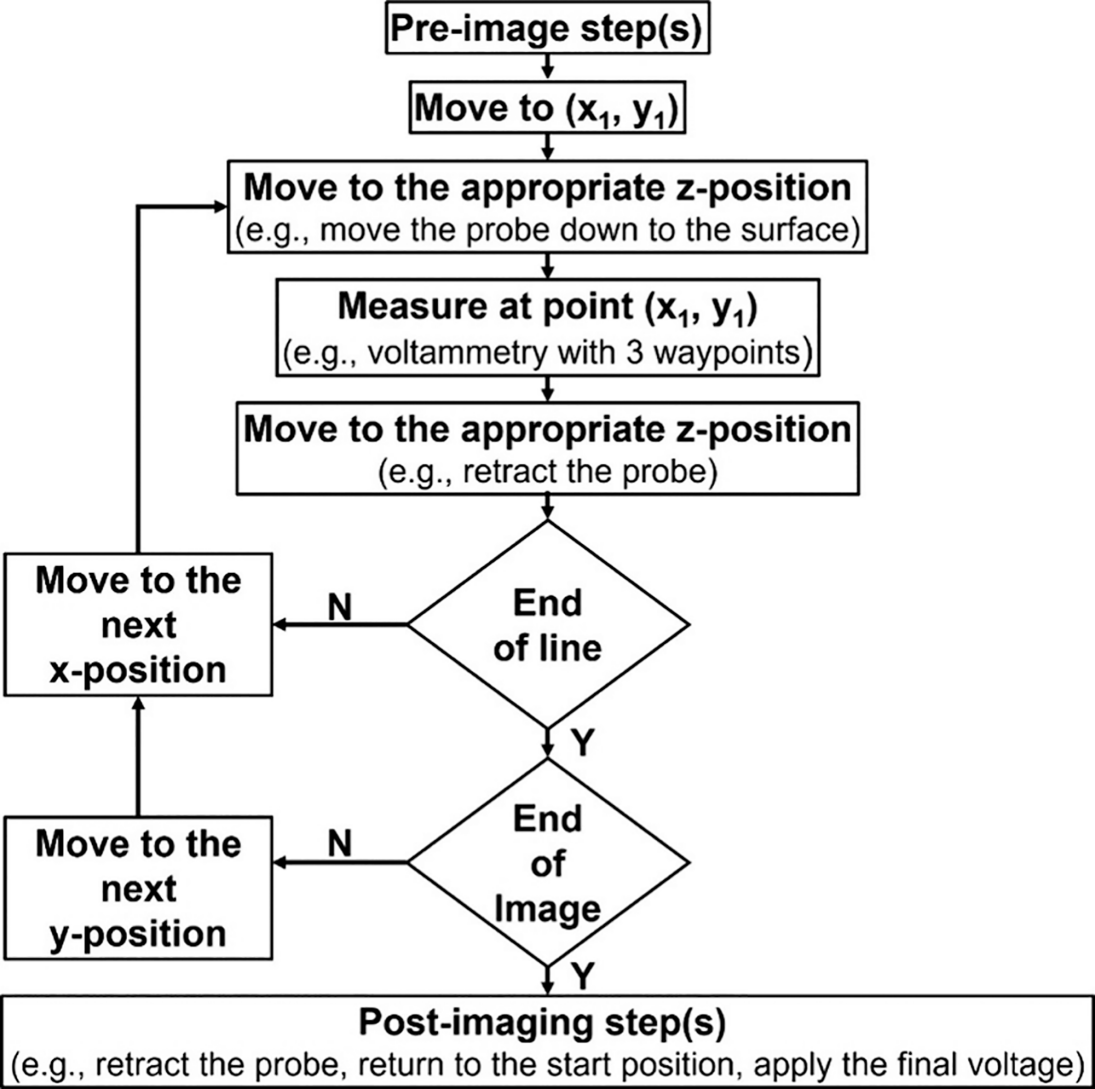
Schematic of the steps taken to generate waypoints for a generic experiment with the hopping scanning mode. Depending on the experiment, each box may represent one, zero, or multiple waypoints.

### Software Architecture

Breaking down an experiment into waypoints means that every experiment comprises the same basic elements: defining a set of waypoints from the user-defined parameters (sweep rate, scan area, applied voltages, etc.) and protocol chosen; performing the instructions encoded by the waypoints; and presenting and storing the data recorded while following the waypoints. To achieve this functionality, the system is separated into multiple layers, each of which interacts with the layers immediately above/below (see Figure 5; detailed description below). The user only interacts with the top layer (Experiment-Specific User Interface), while technical details are handled by lower layers that are abstracted away from the user. The bottom layer is a program that runs on the FPGA-based DAQ card, allowing it to react to stimuli on sub-ms time scales.

**Figure 5:**
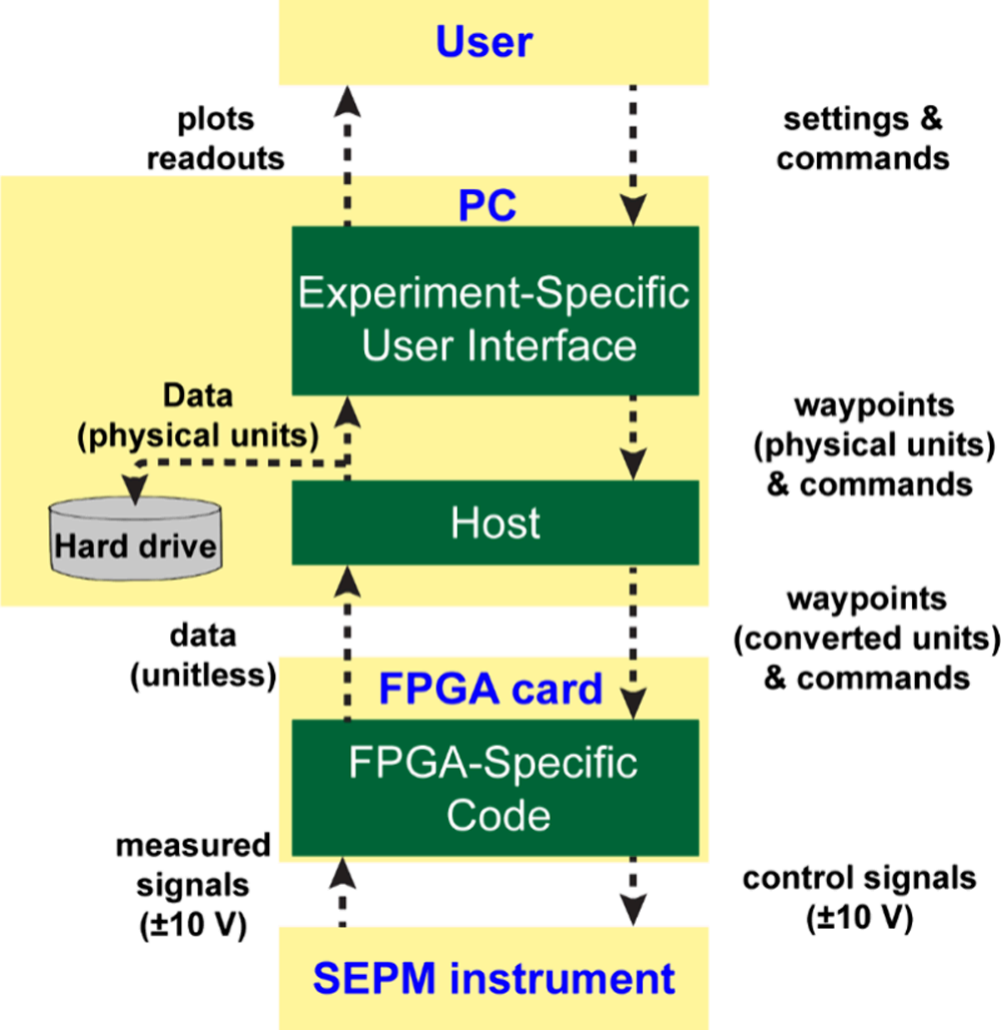
Diagram showing the structure of the software that is used to control the SEPM instrument. Labeled arrows represent the information that is communicated and the direction of communication.

All the software is written in the LabVIEW software, in which each program (VI in LabVIEW parlance) consists of a front panel, which looks like the front of a physical instrument with inputs and outputs such as dials, gauges, graphs, switches, drop-down menus, etc. and a back panel, which is hidden from the user, which encodes how the inputs from the front panel are processed and data output via various programming elements.

### Experiment-Specific User Interface

Experiment-specific user interfaces are programs that run on the PC and enable user interaction with the instrument. *It is the only layer in the hierarchy (* Figure 5 *) that varies from experiment to experiment*; i.e., for each experiment (CV, hopping mode imaging, etc.), a different experiment-specific user interface exists. The front panel of the experiment-specific user interface contains controls that allow the user to define experimental parameters and interact with the experiment as it runs (e.g., stop or pause an experiment, or adjust feedback parameters), and plots and indicators that display experimental data to the user in real-time.

In the back panel, the settings are used to create the waypoints for a specific experiment, which are then passed to the next layer (Host). The interface also provides the Host with references for the graphs and indicators to which data/values are plotted, and to the buttons/controls that allow the user to interact with a running experiment (stop/pause/feedback parameters). The front panel and back panels for an experiment-specific user interface that performs a hopping scan are presented in section S5 of the Supporting Information.

Experiment-specific user interfaces for commonly used SEPM techniques, such as CV, chrono-amperometry/potentiometry, approach curves, constant-distance scanning, hopping-mode scanning, and numerous custom experiments, are provided as part of the software suite.

### Host

The host code is common to all experiments and runs on the PC. The host code translates the waypoints (i.e., changes physical units to voltages; see *Instrument Control and Data Acquisition* for further details) and transfers them to the FPGA-based DAQ card, to which it also relays any changes to buttons/controls that control ‘on-the-fly’ functionality (stop/start/feedback parameters/...). The host code transfers the data collected by the FPGA-based DAQ card to the PC, where it is translated (voltage → physical units) and simultaneously streamed to the hard disk (TDMS file format; see Supporting Information, section S4 for details) and presented to the user through plots and indicators on the experiment-specific interface front panel. While the settings and data that are interchanged vary from experiment to experiment, the job of this code remains the same, and so it does not need to be changed by the user.

### FPGA-Specific Code

The FPGA-specific code is common to all experiments and runs on the FPGA-based DAQ card. It follows the instructions encoded by the waypoints. The different feedback schemes (see *Feedback and Moving between Waypoints*) are coded into the FPGA-specific code, meaning that they can be reused simply by specifying them in the waypoints (see *Experiment-Specific User Interface*), abstracting the user from the complexities of programming this component. The program runs at 80 MHz, meaning that the response to changing signals can be rapid. The FPGA-based DAQ card also collects data and streams it back to the PC via the host.

### Summary

Overall, the code is designed so that new probe movement schemes can be defined either by writing a new experiment-specific user interface or by modifying an existing one, while keeping the Host and FPGA code unchanged. The FPGA-based DAQ card and Host are configured to continuously stream and save data (TDMS and text formats) from all channels (*xyz* position, current, voltage, feedback type, etc.—see Table S1 for a complete list) during the entire experiment. Thus, all the data are always available to the user for inspection, processing, and analysis (see Supporting Information, section S4 for details of the data format). The experiment-specific user interfaces can generate basic plots of the acquired data, while complex analysis of the data can be performed post-experiment using tools of the user’s choosing. Examples of using MATLAB scripts to analyze data are provided in section S6 of the Supporting Information.

### Software Availability

The open-source code is freely available to users from a GitHub repository (see Supporting Information section S7 details). Using a repository allows code updates, new experiments, and improvements to be quickly and easily provided to users.

## Example Experiments

The functionality and flexibility of the instrument described above are well-documented by numerous examples in the literature, spanning SECM,^67,89^ SICM,^51–53,51–53,118–124^ SECCM,^56,59,71,77,92,125–140^ and their hyphened techniques.^65,69,70,74,76,109,114,115^ The uptake of WEC-SPM has been particularly strong in the global development of SECCM and SICM, where applications have expanded to include characterizing the local electrochemical activity of biological samples^141–143^ and analysis of the corrosion behavior of complex metal surfaces.^144–147^ WEC-SPM has enabled single-entity measurements,^129,148^ correlating structure to electrochemical activity,^149,150^ characterization of electrochemical metal deposition and dissolusion,^151–154^ and advanced catalytic activity studies.^127,130,155,156^ It has also been applied to electrochemical nanobubble nucleation and growth, offering insights into gas evolution dynamics at the nanoscale,^157^ and to 3D printing and patterning.^11,158^ Furthermore, WEC-SPM has been integrated with hyphenated techniques, including SECM and interference reflection microscopy, allowing for *in situ* detection of reaction intermediates^69,70^ and the study of droplet behavior,^114^ nanoparticle nucleation, and growth in confined spaces.^76,115^

The flexibility of WEC-SPM-controlled instruments has allowed the adoption of scan patterns beyond conventional raster scanning. One such example is high-speed spiral scanning with a stored trajectory. First, topography is measured and stored within the platform during a slower constant-distance scan (imaging feedback engaged). Next, the stored topography is retraced during high-speed imaging (see Supporting Information, Table S2 for these two modes).^125^ By minimizing both piezo acceleration (spiral pattern) and overcoming the response time of feedback, dramatically enhanced scan speeds (up to 4 seconds per frame; typically 1000 pixels μm^–2^ per frame) have been achieved in both SECCM^125^ and SICM.^53^

These developments highlight the versatility and growing applications of WEC-SPM in diverse fields of electrochemical research. Below, we describe two further examples where SICM is used to characterize nanoparticle growth and reactions on a Au nanocluster. Both examples use the experimental setup shown in Figure 6A, where the potential of the substrate and SICM probe are independently controlled by connecting to a potentiostat, allowing us to control and interrogate electrochemical processes on the substrate (for more details of the instrument, see the Supporting Information, section S8).

**Figure 6:**
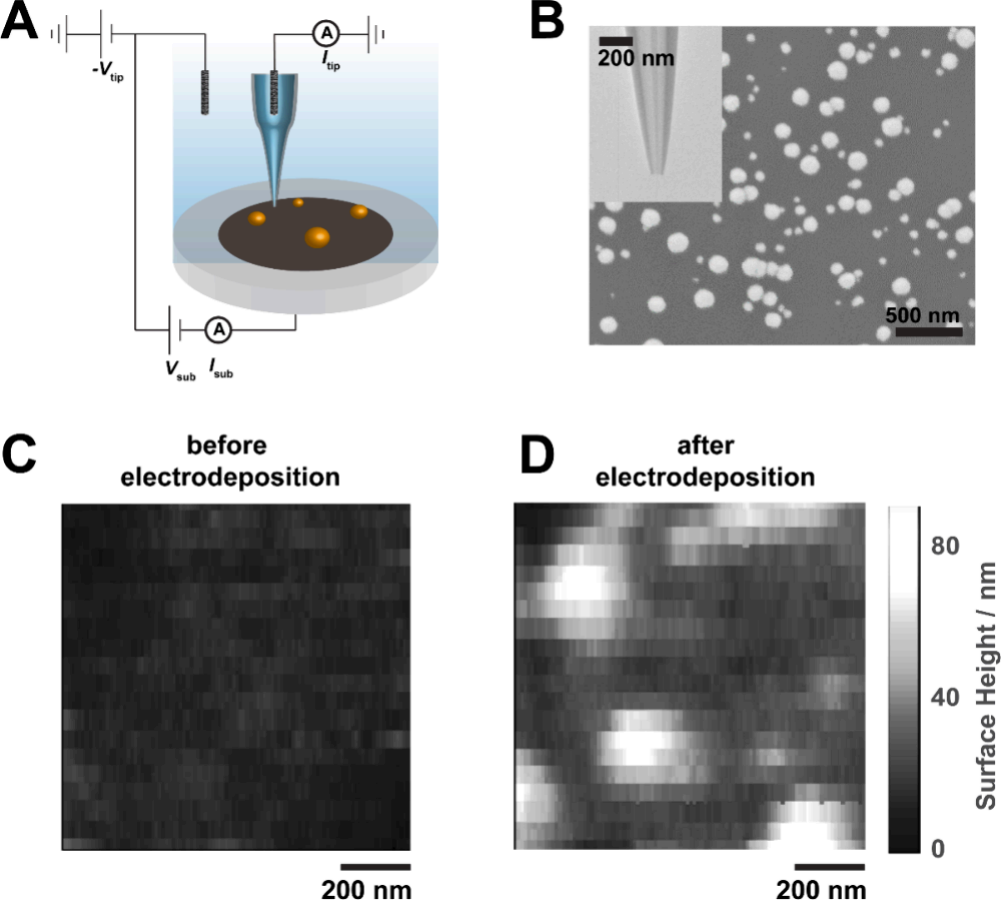
SICM characterization of Pt nanoparticle (PtNP) electrodeposition. (A) Schematic of the experimental setup for SICM with potential control of the substrate. (B) Scanning electron micrograph of carbon substrate with electrodeposited PtNPs. (Inset) SICM probe characterized by TEM (the dark line in the middle of the pipette shows a glass filament, which aids in filling of the pipette through capillary action). SICM topographic images of the polished carbon substrate before (C) and after (D) the electrodeposition of PtNPs (no interpolation is used in these images).

### Example 1 - Constant Distance *in Situ* Topographic Imaging of Nanoparticle Growth

Nanopipettes with an orifice diameter ranging from 30 to 50 nm (Figure 6B, inset) were used as SICM probes to perform high-resolution imaging of the electrodeposition of Pt nanoparticles (PtNPs) on a glassy carbon electrode (see Supporting Information, Section S8 for probe fabrication). Constant-distance imaging with AC feedback, using distance modulation mode,^95^ was used to maintain precise separation between the probe and the substrate (i.e., constant-distance scanning in Figure 2B; see *Operation of an SEPM: Movement schemes*). The probe position was oscillated sinusoidally normal to the substrate electrode with an amplitude of 20 nm at 311 Hz by feeding the signal from a lock-in amplifier and the piezo *z*-position control into a voltage adder, the output of which was then sent to the *z*-input of the piezo controller (see Figure S8 for connections).

With the sample immersed in 100 mM HClO_4_ and 1 mM K_2_PtCl_6_, a topography map was recorded with *V*_sub_ = 0.7 V vs Ag/AgCl wire QRCE (a potential at which Pt was not electrodeposited onto the carbon substrate electrode). The resulting image (Figure 6C) shows the flat glassy carbon surface. Cyclic voltammetry of the carbon substrate was then performed (2 cycles, 0.7 to −0.45 V vs Ag/AgCl wire QRCE, ν = 0.2 V/s), to electrodeposit PtNPs on it, and another topography map of the same region was acquired. Figure 6D shows this *in situ* image, which provides a 3D map of the PtNPs, showing heights ranging from 10 to 80 nm. In addition to demonstrating the high-resolution imaging that can be performed on the instrument, we also see how independent control of the potential of the sample and the tip allows one to correlate the location of electrodeposition to the starting topography. For more examples of *in situ* topographic mapping using the flexible open-source instrument, see references.^11,52,65,118,120,121^

### Example 2 - Hopping Mode for Simultaneous Mapping of Topography and Reactions

Using carefully designed protocols where the tip-surface separation and the potential are manipulated, SICM can also be utilized to investigate a wide variety of physicochemical properties of interfaces, such as surface charge,^51,52,121,159,160^ ion flux, and electrochemical reactions.^53,118,119^ Figure 7 shows the use of SICM to map the topography and local ion flux (Figure 7A) on a gold nanocluster (AuNC; Figure 7B), due to BH_4_^–^ electro-oxidation. Full experimental details can be found in Supporting Information, Section S8. These measurements use the hopping mode scan protocol shown schematically in Figure 7A. Step 1: the SICM probe was moved to ∼6 nm from the surface, as evaluated by a 4.1% decrease in the current at the SICM probe (*I*_tip_) of 4.1 %, compared to the bulk value at the start the approach (i.e., hopping mode scanning in Figure 2C; see *Operation of an SEPM: Movement Schemes*);^65^ this distance provides the sample height used in the topographical map (Figure 7C; see Supporting Information, section S6 for MATLAB script generating the figure). The SICM topography shows the same dimensions of the AuNC as seen in the SEM and provides additional information about its height (∼600 nm). Note that the large aspect ratio and rough structure are easily characterized by hopping imaging, while constant distance imaging of such a sample would risk the tip colliding with the sample. Step 2: the probe was retracted by 5 nm, (“small lift-up” step), which reduces the impact of the probe on mass transport to the substrate and double-layer effects on the *I*_tip_. Step 3: After the small lift-up, the substrate potential was stepped to turn on electro-oxidation of BH_4_^–^ on the AuNC (*V*_sub_: 0.1 V → 1.35 V vs Pd–H_2_ QRCE). The difference in *I*_tip_ before and after the potential switch quantifies the drop in the local ion concentration (conductivity) caused by the oxidation of BH_4_^–^ (BH_4_^–^ + 8OH^–^ → BO_2_^–^ + 6H_2_O + 8e^–^). Step 4 (Figure 7A): Finally, *V*_sub_ was returned to 0.1 V (vs Pd–H_2_ QRCE), the probe was retracted to bulk solution (*z*-position increased by 750 nm), moved to the next *x–y* position, and the procedure was repeated. Each pixel (approach, measurement, retract) took ∼0.3 s, allowing the 2 × 2 μm high-resolution scan (400 pixels μm^–2^) to be completed in approximately 8 min. In Figure 7D we see ion depletion in the vicinity of the AuNC that is visualized through the ratio of the current at 1.35 V (BH_4_^–^ electrooxidation; end of step 3) to that at 0.1 V (no oxidation; end of step 2). The largest depletion (lowest ratio) is at the side of the AuNC, which we attribute to the narrow gap between the AuNC and the substrate, leading to more depletion of reactants.^53,118^ For more examples of using the open-source instrument in SICM-based reaction mapping, see references 53,118,119,122

**Figure 7:**
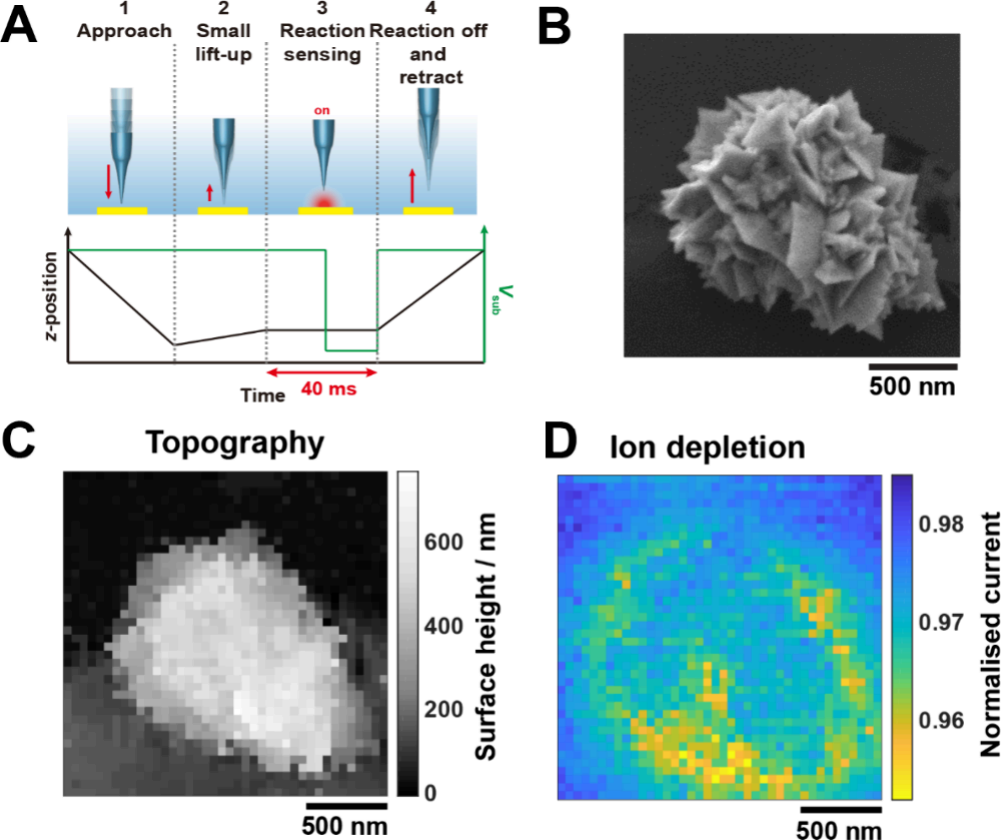
SICM imaging of topography and BH_4_^–^ electrooxidation on a Au nanocluster (AuNC) in 30 mM NaOH (pH 12.5) aqueous solution with 3 mM NaBH_4_. (A) Probe height (black) and substrate potential (green) during ‘small lift-up’ hopping imaging, as used for measuring topography and monitoring reactions through ion-depletion. Reprinted from Ref^118^ with permission from American Chemical Society (ACS). Note that further permissions related to the material excerpted should be directed to the ACS. (B) SEM image of a AuNC. (C) SICM topography and (D) ion depletion maps of the same AuNC. Pipette diameter ∼30 nm. Supporting Information, section S6 shows loading in the data and generation of part C.

## Conclusions

Herein, we described the hardware and software for a flexible open-source, scanning electrochemical probe microscope. This instrument supports a wide range of SEPM techniques (SECM, SICM, SECCM), including hyphenated techniques (e.g., SECM-SICM and SECM-SECCM). Many common imaging protocols are designed into the instrument and custom protocols can be prepared with minimal programming. The flexibility of the instrument means that components can be interchanged to allow different functionality and complementary instrumentation can be interfaced.

Open-source instrumentation (software and hardware), such as described herein, contributes to the larger ecosystem of openness and accessibility in science.^161^ This includes, but is not limited to, openness in data sharing, data handling and analysis tools, and education. Additional contributions in any of these categories could grow and strengthen the field of electrochemical analysis and science in general. Our work contributes to this ecosystem, not only through the above-described instrumentation, but also by making the data from the example measurement available alongside a data analysis script (Supporting Information, Section S6), and through the tutorial introduction provided. We intend for this work to guide and inspire those interested in the potential of SEPM to construct instruments in their own laboratories, and that the flexibility of the instrument will facilitate the development of new imaging protocols and modalities.

## Supplementary Material



## Notes

Parts list, engineering drawings of custom parts and details for fabrication, electrical connections for the instrument, example of a experiment-specific user interface, description of the structure of the data produced, example of a data processing script, details of access to the software and designs, additional experimental details (PDF)

## References

[1] Voigtländer, B. Scanning Probe Microscopy, 1st ed.; Springer: Berlin, Heidelberg, 2015. 10.1007/978-3-662-45240-0.

[2] Chen C., Zhou Y., Baker L. A. (2012). Scanning Ion Conductance Microscopy. Annu. Rev. Anal. Chem..

[3] Polcari D., Dauphin-Ducharme P., Mauzeroll J. (2016). Scanning Electrochemical Microscopy:
A Comprehensive Review of Experimental Parameters from 1989 to 2015. Chem. Rev..

[4] O’Connell M. A., Wain A. J. (2015). Combined Electrochemical-Topographical
Imaging: A Critical
Review. Anal. Methods.

[5] Bentley C. L., Edmondson J., Meloni G. N., Perry D., Shkirskiy V., Unwin P. R. (2019). Nanoscale Electrochemical Mapping. Anal. Chem..

[6] Zoski C. G. (2016). Review—Advances
in Scanning Electrochemical Microscopy (SECM). J. Electrochem. Soc..

[7] Kai T., Zhou M., Johnson S., Ahn H. S., Bard A. J. (2018). Direct
Observation of C_2_O_4_
^•–^ and CO_2_
^•–^ by Oxidation of Oxalate
within Nanogap of Scanning Electrochemical Microscope. J. Am. Chem. Soc..

[8] McGeouch C.-A., Edwards M. A., Mbogoro M. M., Parkinson C., Unwin P. R. (2010). Scanning Electrochemical Microscopy
as a Quantitative
Probe of Acid-Induced Dissolution: Theory and Application to Dental
Enamel. Anal. Chem..

[9] McGeouch C. A., Peruffo M., Edwards M. A., Bindley L. A., Lazenby R. A., Mbogoro M. M., McKelvey K., Unwin P. R. (2012). Quantitative Localized
Proton-Promoted Dissolution Kinetics of Calcite Using Scanning Electrochemical
Microscopy (SECM). J. Phys. Chem. C.

[10] Oseland E.
E., Ayres Z. J., Basile A., Haddleton D. M., Wilson P., Unwin P. R. (2016). Surface
Patterning of Polyacrylamide
Gel Using Scanning Electrochemical Cell Microscopy (SECCM). Chem. Commun..

[11] Momotenko D., Page A., Adobes-Vidal M., Unwin P. R. (2016). Write-Read 3D Patterning
with a Dual-Channel Nanopipette. ACS Nano.

[12] Ebejer N., Schnippering M., Colburn A. W., Edwards M. A., Unwin P. R. (2010). Localized
High Resolution Electrochemistry and Multifunctional Imaging: Scanning
Electrochemical Cell Microscopy. Anal. Chem..

[13] Lohrengel M. M., Moehring A., Pilaski M. (2001). Capillary-Based Droplet Cells: Limits
and New Aspects. Electrochim. Acta.

[14] Williams C. G., Edwards M. A., Colley A. L., Macpherson J. V., Unwin P. R. (2009). Scanning Micropipet Contact Method
for High-Resolution
Imaging of Electrode Surface Redox Activity. Anal. Chem..

[15] Macpherson J. V., Unwin P. R. (2000). Combined Scanning Electrochemical-Atomic Force Microscopy. Anal. Chem..

[16] Kranz C., Friedbacher G., Mizaikoff B., Lugstein A., Smoliner J., Bertagnolli E. (2001). Integrating an Ultramicroelectrode in an AFM Cantilever:
Combined Technology for Enhanced Information. Anal. Chem..

[17] Jayamaha G., Maleki M., Bentley C. L., Kang M. (2024). Practical Guidelines
for the Use of Scanning Electrochemical Cell Microscopy (SECCM). Analyst.

[18] Anderson K. L., Edwards M. A. (2025). A Tutorial for Scanning
Electrochemical Cell Microscopy
(SECCM) Measurements: Step-by-Step Instructions, Visual Resources,
and Guidance for First Experiments. ACS Meas.
Sci. Au.

[19] Santana
Santos C., Jaato B. N., Sanjuán I., Schuhmann W., Andronescu C. (2023). Operando Scanning Electrochemical
Probe Microscopy during Electrocatalysis. Chem.
Rev..

[20] Mishra A., Sarbapalli D., Rodríguez O., Rodríguez-López J. (2023). Electrochemical
Imaging of Interfaces in Energy Storage via Scanning Probe Methods:
Techniques, Applications, and Prospects. Annu.
Rev. Anal. Chem..

[21] Edwards M. A., Martin S., Whitworth A. L., Macpherson J. V., Unwin P. R. (2006). Scanning Electrochemical Microscopy:
Principles and
Applications to Biophysical Systems. Physiol.
Meas..

[22] Conzuelo F., Schulte A., Schuhmann W. (2018). Biological
Imaging with Scanning
Electrochemical Microscopy. Proc. R. Soc. A.

[23] Zhou Y., Takahashi Y., Fukuma T., Matsue T. (2021). Scanning Electrochemical
Microscopy for Biosurface Imaging. Curr. Opin.
Electrochem..

[24] Oja S. M., Fan Y., Armstrong C. M., Defnet P., Zhang B. (2016). Nanoscale Electrochemistry
Revisited. Anal. Chem..

[25] Baker L. A. (2018). Perspective
and Prospectus on Single-Entity Electrochemistry. J. Am. Chem. Soc..

[26] Wahab O. J., Kang M., Unwin P. R. (2020). Scanning
Electrochemical Cell Microscopy:
A Natural Technique for Single Entity Electrochemistry. Curr. Opin. Electrochem..

[27] Edwards M. A., Robinson D. A., Ren H., Cheyne C. G., Tan C. S., White H. S. (2018). Nanoscale Electrochemical
Kinetics & Dynamics:
The Challenges and Opportunities of Single-Entity Measurements. Faraday Discuss..

[28] WEC-SPM Academic Use License. https://warwick.ac.uk/fac/sci/chemistry/research/unwin/electrochemistry/wec-spm/academic_use__licence_warwick_electrochemical_scanning_probe_microscopy_wec-spm.pdf (accessed 2025-07-22).

[29] Valtr M., Klapetek P., Martinek J., Novotný O., Jelínek Z., Hortvík V., Nečas D. (2023). Scanning Probe
Microscopy Controller with Advanced Sampling Support. HardwareX.

[30] Adams J. D., Nievergelt A., Erickson B. W., Yang C., Dukic M., Fantner G. E. (2014). High-Speed Imaging Upgrade for a
Standard Sample Scanning
Atomic Force Microscope Using Small Cantilevers. Rev. Sci. Instrum..

[31] Zahl P., Bierkandt M., Schröder S., Klust A. (2003). The Flexible and Modern
Open Source Scanning Probe Microscopy Software Package GXSM. Rev. Sci. Instrum..

[32] Guver A., Fifita N., Milas P., Straker M., Guy M., Green K., Yildirim T., Unlu I., Yigit M. V., Ozturk B. (2019). A Low-Cost and High-Precision Scanning Electrochemical
Microscope Built with Open Source Tools. HardwareX.

[33] Meloni G. N. (2017). 3D Printed
and Microcontrolled: The One Hundred Dollars Scanning Electrochemical
Microscope. Anal. Chem..

[34] Binnig G., Rohrer H. (1983). Scanning Tunneling
Microscopy. Surf. Sci..

[35] Bard A. J., Fan F. F., Kwak J., Lev O. (1989). Scanning Electrochemical
Microscopy. Introduction and Principles. Anal.
Chem..

[36] Engstrom R. C. (1984). Spatial
Resolution of Electrode Heterogeneity Using Iontophoresis. Anal. Chem..

[37] Engstrom R. C., Weber M., Werth J. (1985). Distribution of Electrochemical Activity
on Graphite-Epoxy Surfaces. Anal. Chem..

[38] Engstrom R. C., Weber M., Wunder D. J., Burgess R., Winquist S. (1986). Measurements
within the Diffusion Layer Using a Microelectrode Probe. Anal. Chem..

[39] Amemiya S., Bard A. J., Fan F. R. F., Mirkin M. V., Unwin P. R. (2008). Scanning
Electrochemical Microscopy. Annu. Rev. Anal.
Chem..

[40] Bard A. J., Fan F.-R. F., Pierce D. T., Unwin P. R., Wipf D. O., Zhou F. (1991). Chemical Imaging of
Surfaces with the Scanning Electrochemical Microscope. Science.

[41] Sánchez-Sánchez C. M., Rodríguez-López J., Bard A. J. (2008). Scanning Electrochemical
Microscopy. 60. Quantitative Calibration of the SECM Substrate Generation/Tip
Collection Mode and Its Use for the Study of the Oxygen Reduction
Mechanism. Anal. Chem..

[42] Liu B., Bard A. J. (2002). Scanning Electrochemical
Microscopy. 45. Study of the
Kinetics of Oxygen Reduction on Platinum with Potential Programming
of the Tip. J. Phys. Chem. B.

[43] Fernández J. L., Bard A. J. (2004). Scanning Electrochemical
Microscopy 50. Kinetic Study
of Electrode Reactions by the Tip Generation-Substrate Collection
Mode. Anal. Chem..

[44] Unwin P. R., Bard A. J. (1992). Scanning Electrochemical
Microscopy. 14. Scanning Electrochemical
Microscope Induced Desorption: A New Technique for the Measurement
of Adsorption/Desorption Kinetics and Surface Diffusion Rates at the
Solid/Liquid Interface. J. Phys. Chem..

[45] Zhang J., Slevin C. J., Morton C., Scott P., Walton D. J., Unwin P. R. (2001). New Approach for Measuring Lateral Diffusion in Langmuir
Monolayers by Scanning Electrochemical Microscopy (SECM): Theory and
Application. J. Phys. Chem. B.

[46] O’Mullane A. P., Macpherson J. V., Unwin P. R., Cervera-Montesinos J., Manzanares J. A., Frehill F., Vos J. G. (2004). Measurement of Lateral
Charge Propagation in [Os­(Bpy)_2_(PVP)_n_Cl]Cl Thin
Films: A Scanning Electrochemical Microscopy Approach. J. Phys. Chem. B.

[47] Rodríguez-López J., Alpuche-Avilés M. A., Bard A. J. (2008). Interrogation of
Surfaces for the Quantification of Adsorbed Species on Electrodes:
Oxygen on Gold and Platinum in Neutral Media. J. Am. Chem. Soc..

[48] Hansma P. K., Drake B., Marti O., Gould S. A. C., Prater C. B. (1989). The Scanning
Ion-Conductance Microscope. Science.

[49] Zhu C., Huang K., Siepser N. P., Baker L. A. (2021). Scanning Ion Conductance
Microscopy. Chem. Rev..

[50] Page A., Perry D., Unwin P. R. (2017). Multifunctional
Scanning Ion Conductance
Microscopy. Proc. R. Soc. A.

[51] McKelvey K., Perry D., Byers J. C., Colburn A. W., Unwin P. R. (2014). Bias Modulated
Scanning Ion Conductance Microscopy. Anal. Chem..

[52] McKelvey K., Kinnear S. L., Perry D., Momotenko D., Unwin P. R. (2014). Surface Charge Mapping with a Nanopipette. J. Am. Chem. Soc..

[53] Momotenko D., McKelvey K., Kang M., Meloni G. N., Unwin P. R. (2016). Simultaneous
Interfacial Reactivity and Topography Mapping with Scanning Ion Conductance
Microscopy. Anal. Chem..

[54] Suter T., Böhni H. (1997). A New Microelectrochemical
Method to Study Pit Initiation
on Stainless Steels. Elecrochim. Acta.

[55] Eng L., Wirth E., Suter T., Böhni H. (1998). Non-Contact
Feedback for Scanning Capillary Microscopy. Electrochim. Acta.

[56] Ebejer N., Güell A. G., Lai S. C. S., McKelvey K., Snowden M. E., Unwin P. R. (2013). Scanning Electrochemical Cell Microscopy:
A Versatile
Technique for Nanoscale Electrochemistry and Functional Imaging. Annu. Rev. Anal. Chem..

[57] Bentley C. L., Kang M., Unwin P. R. (2017). Nanoscale
Structure Dynamics within
Electrocatalytic Materials. J. Am. Chem. Soc..

[58] Varhade S., Meloni G., Tetteh E. B., Kim M., Schumacher S., Quast T., Andronescu C., Unwin P. R., Schuhmann W. (2023). Elucidation
of Alkaline Electrolyte-Surface Interaction in SECCM Using a pH-Independent
Redox Probe. Electrochim. Acta.

[59] Kinnear S. L., McKelvey K., Snowden M. E., Peruffo M., Colburn A. W., Unwin P. R. (2013). Dual-Barrel Conductance
Micropipet as a New Approach
to the Study of Ionic Crystal Dissolution Kinetics. Langmuir.

[60] Parker A. S., Al Botros R., Kinnear S. L., Snowden M. E., McKelvey K., Ashcroft A. T., Carvell M., Joiner A., Peruffo M., Philpotts C., Unwin P. R. (2016). Combinatorial Localized Dissolution
Analysis: Application to Acid-Induced Dissolution of Dental Enamel
and the Effect of Surface Treatments. J. Colloid
Interface Sci..

[61] Patel A. N., Collignon M. G., O’Connell M. A., Hung W. O. Y., McKelvey K., Macpherson J. V., Unwin P. R. (2012). A New View of Electrochemistry at
Highly Oriented Pyrolytic Graphite. J. Am. Chem.
Soc..

[62] Mariano R. G., McKelvey K., White H. S., Kanan M. W. (2017). Selective Increase
in CO_2_ Electroreduction Activity at Grain-Boundary Surface
Terminations. Science.

[63] Anderson K. L., Edwards M. A. (2023). Evaluating Analytical
Expressions for Scanning Electrochemical
Cell Microscopy (SECCM). Anal. Chem..

[64] Takahashi Y., Shevchuk A. I., Novak P., Murakami Y., Shiku H., Korchev Y. E., Matsue T. (2010). Simultaneous Noncontact Topography
and Electrochemical Imaging by SECM/SICM Featuring Ion Current Feedback
Regulation. J. Am. Chem. Soc..

[65] Page A., Kang M., Armitstead A., Perry D., Unwin P. R. (2017). Quantitative
Visualization of Molecular Delivery and Uptake at Living Cells with
Self-Referencing Scanning Ion Conductance Microscopy-Scanning Electrochemical
Microscopy. Anal. Chem..

[66] Comstock D. J., Elam J. W., Pellin M. J., Hersam M. C. (2010). Integrated Ultramicroelectrode-Nanopipet
Probe for Concurrent Scanning Electrochemical Microscopy and Scanning
Ion Conductance Microscopy. Anal. Chem..

[67] McKelvey K., Nadappuram B. P., Actis P., Takahashi Y., Korchev Y. E., Matsue T., Robinson C., Unwin P. R. (2013). Fabrication,
Characterization, and Functionalization of Dual Carbon Electrodes
as Probes for Scanning Electrochemical Microscopy (SECM). Anal. Chem..

[68] Liljeroth P., Johans C., Slevin C. J., Quinn B. M., Kontturi K. (2002). Disk-Generation/Ring-Collection
Scanning Electrochemical Microscopy: Theory and Application. Anal. Chem..

[69] Paulose
Nadappuram B., McKelvey K., Byers J. C., Güell A. G., Colburn A. W., Lazenby R. A., Unwin P. R. (2015). Quad-Barrel Multifunctional
Electrochemical and Ion Conductance Probe for Voltammetric Analysis
and Imaging. Anal. Chem..

[70] Ryu C. H., Ren H. (2024). Simultaneous Mapping
of Electrocatalytic Activity and Selectivity
via Hybrid Scanning Electrochemical Probe Microscopy. Nano Lett..

[71] Zerdoumi R., Quast T., Tetteh E. B., Kim M., Li L., Dieckhöfer S., Schuhmann W. (2024). Integration
of Scanning Electrochemical
Microscopy and Scanning Electrochemical Cell Microscopy in a Bifunctional
Nanopipette toward Simultaneous Mapping of Activity and Selectivity
in Electrocatalysis. Anal. Chem..

[72] Saha-Shah A., Weber A. E., Karty J. A., Ray S. J., Hieftje G. M., Baker L. A. (2015). Nanopipettes : Probes for Local Sample Analysis. Chem. Sci..

[73] Momotenko D., Qiao L., Cortés-Salazar F., Lesch A., Wittstock G., Girault H. H. (2012). Electrochemical Push-Pull Scanner
with Mass Spectrometry Detection. Anal. Chem..

[74] Zhang L., Edwards M. E., Wahab O. J., Samayoa-Oviedo H. Y., Freitas D. P., Yan X., Baker L. A. (2025). Scanning
Electrochemical
Cell Microscopy for Sub-Micrometer Mass Spectrometric Studies of Electrochemical
Reactions. ACS Electrochem..

[75] Korchev Y. E., Raval M., Gorelik J., Edwards C. R. W., Rayment T. (2000). Hybrid Scanning
Ion Conductance and Scanning Near-Field Optical Microscopy for the
Study of Living Cells. Biophys. J..

[76] Ciocci P., Valavanis D., Meloni G. N., Lemineur J., Unwin P. R., Kanoufi F. (2023). Optical Super-Localisation of Single Nanoparticle Nucleation
and Growth in Nanodroplets. ChemElectroChem.

[77] Tetteh E. B., Valavanis D., Daviddi E., Xu X., Santana
Santos C., Ventosa E., Martín-Yerga D., Schuhmann W., Unwin P. R. (2023). Fast Li-Ion Storage and Dynamics
in TiO_2_ Nanoparticle Clusters Probed by Smart Scanning
Electrochemical Cell Microscopy. Angew. Chem.,
Int. Ed..

[78] Lee Y., Ding Z., Bard A. J. (2002). Combined Scanning Electrochemical/Optical
Microscopy with Shear Force and Current Feedback. Anal. Chem..

[79] Macpherson J. V., Unwin P. R., Hillier A. C., Bard A. J. (1996). In-Situ Imaging
of Ionic Crystal Dissolution Using an Integrated Electrochemical/AFM
Probe. J. Am. Chem. Soc..

[80] Patel A. N., Kranz C. (2018). (Multi)­Functional Atomic
Force Microscopy Imaging. Annu. Rev. Anal. Chem..

[81] Kai T., Zoski C. G., Bard A. J. (2018). Scanning
Electrochemical Microscopy
at the Nanometer Level. Chem. Commun..

[82] Fernández J. L., Walsh D. A., Bard A. J. (2005). Thermodynamic Guidelines for the
Design of Bimetallic Catalysts for Oxygen Electroreduction and Rapid
Screening by Scanning Electrochemical Microscopy. M-Co (M: Pd, Ag,
Au). J. Am. Chem. Soc..

[83] Takahashi Y., Shevchuk A. I., Novak P., Babakinejad B., Macpherson J. V., Unwin P. R., Shiku H., Gorelik J., Klenerman D., Korchev Y. E., Matsue T. (2012). Topographical
and Electrochemical
Nanoscale Imaging of Living Cells Using Voltage-Switching Mode Scanning
Electrochemical Microscopy. Proc. Natl. Acad.
Sci. U.S.A..

[84] Eckhard K., Schuhmann W. (2008). Alternating Current Techniques in Scanning Electrochemical
Microscopy (AC-SECM). Analyst.

[85] Edwards M.
A., Whitworth A. L., Unwin P. R. (2011). Quantitative Analysis and Application
of Tip Position Modulation-Scanning Electrochemical Microscopy. Anal. Chem..

[86] Ludwig M., Kranz C., Schuhmann W., Gaub H. E. (1995). Topography Feedback
Mechanism for the Scanning Electrochemical Microscope Based on Hydrodynamic
Forces between Tip and Sample. Revi. Sci. Instrum..

[87] Knittel P., Mizaiko B., Kranz C. (2016). Simultaneous
Nanomechanical and Electrochemical
Mapping: Combining Peak Force Tapping Atomic Force Microscopy with
Scanning Electrochemical Microscopy. Anal. Chem..

[88] Cortés-Salazar F., Momotenko D., Lesch A., Wittstock G., Girault H. H. (2010). Soft Microelectrode
Linear Array for Scanning Electrochemical
Microscopy. Anal. Chem..

[89] McKelvey K., Edwards M. A., Unwin P. R. (2010). Intermittent
Contact-Scanning Electrochemical
Microscopy (IC-SECM): A New Approach for Tip Positioning and Simultaneous
Imaging of Interfacial Topography and Activity. Anal. Chem..

[90] Alpuche-Aviles M. A., Wipf D. O. (2001). Impedance Feedback Control for Scanning Electrochemical
Microscopy. Anal. Chem..

[91] Novak P., Li C., Shevchuk A. I., Stepanyan R., Caldwell M., Hughes S., Smart T. G., Gorelik J., Ostanin V. P., Lab M. J., Moss G. W. J., Frolenkov G. I., Klenerman D., Korchev Y. E. (2009). Nanoscale Live-Cell
Imaging Using Hopping Probe Ion
Conductance Microscopy. Nat. Methods.

[92] Aaronson B.
D. B., Byers J. C., Colburn A. W., McKelvey K., Unwin P. R. (2015). Scanning
Electrochemical Cell Microscopy Platform for Ultrasensitive Photoelectrochemical
Imaging. Anal. Chem..

[93] Happel P., Dietzel I. D. (2009). Backstep Scanning
Ion Conductance Microscopy as a Tool
for Long Term Investigation of Single Living Cells. J. Nanobiotechnol..

[94] Happel P., Hoffmann G., Mann S. A., Dietzel I. D. (2003). Monitoring Cell
Movements and Volume Changes with Pulse-Mode Scanning Ion Conductance
Microscopy. J. Microsc..

[95] Li C., Johnson N., Ostanin V., Shevchuk A., Ying L., Korchev Y., Klenerman D. (2008). High Resolution
Imaging Using Scanning
Ion Conductance Microscopy with Improved Distance Feedback Control. Prog. Nat. Sci..

[96] Shevchuk A. I., Gorelik J., Harding S. E., Lab M. J., Klenerman D., Korchev Y. E. (2001). Simultaneous Measurement
of Ca^2+^ and Cellular
Dynamics: Combined Scanning Ion Conductance and Optical Microscopy
to Study Contracting Cardiac Myocytes. Biophys.
J..

[97] Bentley C. L., Perry D., Unwin P. R. (2018). Stability and Placement
of Ag/AgCl
Quasi-Reference Counter Electrodes in Confined Electrochemical Cells. Anal. Chem..

[98] Gerroll B. H. R., Kulesa K. M., Ault C. A., Baker L. A. (2023). Legion: An Instrument
for High-Throughput Electrochemistry. ACS Meas.
Sci. Au.

[99] Alden S. E., Zhang L., Wang Y., Lavrik N. V., Thorgaard S. N., Baker L. A. (2024). High-Throughput
Single-Entity Electrochemistry with
Microelectrode Arrays. Anal. Chem..

[100] Glasscott M. W., Brown E. W., Dorsey K., Laber C. H., Conley K., Ray J. D., Moores L. C., Netchaev A. (2022). Selecting
an Optimal Faraday Cage to Minimize Noise in Electrochemical Experiments. Anal. Chem..

[101] Kim J., Shen M., Nioradze N., Amemiya S. (2012). Stabilizing Nanometer
Scale Tip-to-Substrate Gaps in Scanning Electrochemical Microscopy
Using an Isothermal Chamber for Thermal Drift Suppression. Anal. Chem..

[102] Bard A. J., Wipf D. O. (1992). Scanning Electrochemical
Microscopy.
15. Improvements in Imaging via Tip-Position Modulation and Lock-in
Detection. Anal. Chem..

[103] Korchev Y. E., Bashford C. L., Milovanovic M., Vodyanoy I., Lab M. J. (1997). Scanning Ion Conductance Microscopy
of Living Cells. Biophys. J..

[104] Lai S. C. S., Dudin P. V., MacPherson J. V., Unwin P. R. (2011). Visualizing Zeptomole (Electro)­Catalysis at Single
Nanoparticles within an Ensemble. J. Am. Chem.
Soc..

[105] Goines S., Deng M., Glasscott M. W., Leung J. W. C., Dick J. E. (2022). Enhancing
Scanning Electrochemical
Microscopy’s Potential to Probe Dynamic Co-Culture Systems
via Hyperspectral Assisted-Imaging. Analyst.

[106] Saha P., Hill J. W., Walmsley J. D., Hill C. M. (2018). Probing
Electrocatalysis at Individual Au Nanorods via Correlated Optical
and Electrochemical Measurements. Anal. Chem..

[107] Gorelik J., Shevchuk A., Ramalho M., Elliott M., Lei C., Higgins C. F., Lab M. J., Klenerman D., Krauzewicz N., Korchev Y. (2002). Scanning Surface Confocal Microscopy
for Simultaneous Topographical and Fluorescence Imaging: Application
to Single Virus-like Particle Entry into a Cell. Proc. Natl. Acad. Sci. U. S. A..

[108] Bioanalytical Reviews: Scanning Ion Conductance Microscopy; Schäffer, T. E. , Ed.; Springer: 2022; Vol. 3.

[109] Guerret-Legras L., Audibert J. F., Gonzalez-Ojeda I. M., Dubacheva G. V., Clavier G., Miomandre F. (2020). Time-Resolved
Fluorescence Microscopy Combined with Scanning Electrochemical Microscopy:
A New Way to Visualize Photo-Induced Electron Transfer Quenching with
an Electrofluorochromic Probe. J. Phys. Chem.
C.

[110] Song Q., Alvarez-Laviada A., Schrup S. E., Reilly-O’Donnell B., Entcheva E., Gorelik J. (2023). Opto-SICM Framework Combines Optogenetics
with Scanning Ion Conductance Microscopy for Probing Cell-to-Cell
Contacts. Commun. Biol..

[111] Snowden M. E., Güell A. G., Lai S. C. S., McKelvey K., Ebejer N., O’Connell M. A., Colburn A. W., Unwin P. R. (2012). Scanning
Electrochemical Cell Microscopy: Theory and Experiment for Quantitative
High Resolution Spatially-Resolved Voltammetry and Simultaneous Ion-Conductance
Measurements. Anal. Chem..

[112] Guerret-Legras L., Audibert J. F., Ojeda I. M. G., Dubacheva G. V., Miomandre F. (2019). Combined SECM-Fluorescence Microscopy
Using a Water-Soluble
Electrofluorochromic Dye as the Redox Mediator. Electrochim. Acta.

[113] Salamifar S. E., Lai R. Y. (2013). Use of Combined
Scanning Electrochemical
and Fluorescence Microscopy for Detection of Reactive Oxygen Species
in Prostate Cancer Cells. Anal. Chem..

[114] Valavanis D., Ciocci P., McPherson I. J., Meloni G. N., Lemineur J.-F., Kanoufi F., Unwin P. R. (2025). Operando
Electrochemical and Optical Characterization of the Meniscus of Scanning
Electrochemical Cell Microscopy (SECCM) Probes. ACS Electrochem..

[115] Valavanis D., Ciocci P., Meloni G. N., Morris P., Lemineur J. F., McPherson I. J., Kanoufi F., Unwin P. R. (2022). Hybrid
Scanning Electrochemical Cell Microscopy-Interference Reflection Microscopy
(SECCM-IRM): Tracking Phase Formation on Surfaces in Small Volumes. Faraday Discuss..

[116] Daviddi E., Shkirskiy V., Kirkman P. M., Robin M. P., Bentley C. L., Unwin P. R. (2021). Nanoscale Electrochemistry in a Copper/Aqueous/Oil
Three-Phase System: Surface Structure-Activity-Corrosion Potential
Relationships. Chem. Sci..

[117] German S. R., Edwards M. A., Ren H., White H. S. (2018). Critical
Nuclei Size, Rate, and Activation Energy of H_2_ Gas Nucleation. J. Am. Chem. Soc..

[118] Kang M., Perry D., Bentley C. L., West G., Page A., Unwin P. R. (2017). Simultaneous Topography and Reaction
Flux Mapping at and around Electrocatalytic Nanoparticles. ACS Nano.

[119] Chen B., Perry D., Teahan J., McPherson I., Edmondson J., Kang M., Valavanis D., Frenguelli B., Unwin P. R. (2021). Artificial Synapse: Spatiotemporal
Heterogeneities in Dopamine Electrochemistry at a Carbon Fiber Ultramicroelectrode. ACS Meas. Sci. Au.

[120] Perry D., Page A., Chen B., Frenguelli B. G., Unwin P. R. (2017). Differential-Concentration Scanning
Ion Conductance
Microscopy. Anal. Chem..

[121] Page A., Perry D., Young P., Mitchell D., Frenguelli B. G., Unwin P. R. (2016). Fast Nanoscale Surface
Charge Mapping
with Pulsed-Potential Scanning Ion Conductance Microscopy. Anal. Chem..

[122] Chen B., Perry D., Page A., Kang M., Unwin P. R. (2019). Scanning Ion Conductance Microscopy:
Quantitative Nanopipette
Delivery-Substrate Electrode Collection Measurements and Mapping. Anal. Chem..

[123] Ida H., Takahashi Y., Kumatani A., Shiku H., Matsue T. (2017). High Speed
Scanning Ion Conductance Microscopy for Quantitative Analysis of Nanoscale
Dynamics of Microvilli. Anal. Chem..

[124] Wang Y., Shashishekar M., Spence D. M., Baker L. A. (2025). Subcellular
Mechanical Imaging of Erythrocytes with Optically Correlated Scanning
Ion Conductance Microscopy. ACS Meas. Sci.
Au.

[125] Momotenko D., Byers J., McKelvey K., Kang M., Unwin P. R. (2015). High-Speed Electrochemical Imaging. ACS Nano.

[126] Chen C.-H., Jacobse L., McKelvey K., Lai S. C. S., Koper M. T. M., Unwin P. R. (2015). Voltammetric Scanning
Electrochemical
Cell Microscopy: Dynamic Imaging of Hydrazine Electro-Oxidation on
Platinum Electrodes. Anal. Chem..

[127] Harris-Lee T. R., Turvey T., Jayamaha G., Kang M., Marken F., Johnson A. L., Zhang J., Bentley C. L. (2024). Optimizing
Amorphous Molybdenum Sulfide Thin Film Electrocatalysts: Trade-Off
between Specific Activity and Microscopic Porosity. ACS Appl. Mater. Interfaces.

[128] Lee H., Matthews K. C., Zhan X., Warner J. H., Ren H. (2023). Precision
Synthesis of Bimetallic Nanoparticles via Nanofluidics in Nanopipets. ACS Nano.

[129] Kang M., Bentley C. L., Mefford J. T., Chueh W. C., Unwin P. R. (2023). Multiscale Analysis of Electrocatalytic
Particle Activities:
Linking Nanoscale Measurements and Ensemble Behavior. ACS Nano.

[130] Wang Y., Gordon E., Ren H. (2020). Mapping the
Potential
of Zero Charge and Electrocatalytic Activity of Metal-Electrolyte
Interface via a Grain-by-Grain Approach. Anal.
Chem..

[131] Salek S., Byers J. C. (2024). Influence of Particle Size on Mass
Transport during the Oxygen Reduction Reaction at Single Silver Particles
Using Scanning Electrochemical Cell Microscopy. J. Phys. Chem. Lett..

[132] Prabhakaran V., Strange L., Kalsar R., Marina O. A., Upadhyay P., Joshi V. V. (2023). Investigating Electrochemical
Corrosion
at Mg Alloy-Steel Joint Interface Using Scanning Electrochemical Cell
Impedance Microscopy (SECCIM). Sci. Rep..

[133] de Oliveira G. A., Sanchez D. E., Sredenschek A. J., Fest A., Kim M., Santana Santos C., Terrones M., Schuhmann W., Grasseschi D. (2025). Insight into
Mo_2_C Nanoplates Growth by Means of Scanning Electrochemical
Cell Microscopy for High-Resolution Electrocatalytic Hydrogen Evolution
Activity Mapping. Small Sci..

[134] Saleh S., Daboss S., Philipp T., Schäfer D., Rohnke M., Kranz C. (2025). Probing the Properties
of Locally
Formed Solid Electrolyte Interphases on Hard Carbon Anodes. ChemElectroChem.

[135] Takahashi Y., Kobayashi Y., Wang Z., Ito Y., Ota M., Ida H., Kumatani A., Miyazawa K., Fujita T., Shiku H., Korchev Y. E., Miyata Y., Fukuma T., Chen M., Matsue T. (2020). High-Resolution Electrochemical Mapping
of the Hydrogen Evolution Reaction on Transition-Metal Dichalcogenide
Nanosheets. Angew. Chem., Int. Ed..

[136] Zhao J., Wang M., Peng Y., Ni J., Hu S., Zeng J., Chen Q. (2023). Exploring the Strain Effect in Single
Particle Electrochemistry Using Pd Nanocrystals. Angew. Chem., Int. Ed..

[137] Liu Y., Jin C., Liu Y., Ruiz K. H., Ren H., Fan Y., White H. S., Chen Q. (2021). Visualization and Quantification
of Electrochemical H_2_ Bubble Nucleation at Pt, Au, and
MoS_2_ Substrates. ACS Sens..

[138] Zhang H., Tao Y., Pan L., Wang Y., Lee H., Zhan X., Ren H. (2025). Defect-Driven Electrochemical Domain
Modulation in Prussian Blue Revealed by Single-Entity Analysis. J. Am. Chem. Soc..

[139] Takahashi Y., Kumatani A., Munakata H., Inomata H., Ito K., Ino K., Shiku H., Unwin P. R., Korchev Y. E., Kanamura K., Matsue T. (2014). Nanoscale Visualization of Redox
Activity at Lithium-Ion Battery Cathodes. Nat.
Commun..

[140] Zheng Q., Zhuang J., Wang J., Liao X., Meng Y. (2025). Scanning Electrochemical Cell Microscopy
(SECCM) with Hydrogel Probes:
A Theoretical and Experimental Investigation. Anal. Chem..

[141] Wright I. R., Gaudin L. F., Martin L. L., Bentley C. L. (2023). Spatially-Resolved
Bioelectrochemistry with Scanning Electrochemical Cell Microscopy:
A Microscale Study of Coenzyme Q10 Modified Carbon Electrodes. Electrochim. Acta.

[142] Muhammed Y., De Sabatino M., Lazenby R. A. (2025). The Heterogeneity
in the Response of A549 Cells to Toyocamycin Observed Using Hopping
Scanning Ion Conductance Microscopy. J. Phys.
Chem. B.

[143] Muhammed Y., Lazenby R. A. (2024). Scanning Ion Conductance Microscopy
Revealed Cisplatin-Induced Morphological Changes Related to Apoptosis
in Single Adenocarcinoma Cells. Anal. Methods.

[144] Jayamaha G., Tegg L., Bentley C. L., Kang M. (2024). High Throughput
Correlative Electrochemistry-Microscopy Analysis on a Zn-Al Alloy. ACS Phys. Chem. Au.

[145] Coelho L. B., Torres D., Bernal M., Paldino G. M., Bontempi G., Ustarroz J. (2023). Probing the Randomness
of the Local
Current Distributions of 316 L Stainless Steel Corrosion in NaCl Solution. Corros. Sci..

[146] Wang Y., Li M., Gordon E., Ye Z., Ren H. (2022). Nanoscale Colocalized
Electrochemical and Structural Mapping of Metal
Dissolution Reaction. Anal. Chem..

[147] Coelho L. B., Torres D., Vangrunderbeek V., Bernal M., Paldino G. M., Bontempi G., Ustarroz J. (2023). Estimating
Pitting Descriptors of 316 L Stainless Steel by Machine Learning and
Statistical Analysis. Npj Mater. Degrad..

[148] Brunet Cabré M., Schröder C., Pota F., de Oliveira M. A. C., Nolan H., Henderson L., Brazel L., Spurling D., Nicolosi V., Martinuz P., Longhi M., Amargianou F., Bärmann P., Petit T., McKelvey K., Colavita P. E. (2025). Carbon
Thin-Film Electrodes as High-Performing Substrates for Correlative
Single Entity Electrochemistry. Small Methods.

[149] Swan H. B., Gaudin L. F., Hunt A. J., Bentley C. L. (2025). Intrinsic
Electrocatalytic Activity of Platinum Grain Boundaries: Correcting
Measurement Artefacts in Scanning Electrochemical Cell Microscopy
(SECCM). Chem. Sci..

[150] Janjani P., Swan H. B., Dy L., Sarkar A., Bentley C. L. (2025). Enabling
Alkaline Scanning Electrochemical Cell Microscopy
(SECCM) for the Study of Water-Splitting Electrocatalysts. Anal. Chem..

[151] Mondaca-Medina I. E., Ren H. (2024). Site-Specific Stochastic
Rates and
Energetics of Ag Nucleation on Highly Ordered Pyrolytic Graphite. ACS Nano.

[152] Lee H., Zhan X., Warner J. H., Ren H. (2025). Dynamic Ionic Environment
Modulation for Precise Electrosynthesis of Heterostructured Bimetallic
Nanoparticles. Adv. Sci..

[153] Lee H., Muñoz-Castañeda J. A., Ren H. (2025). Facet-Controlled Electrosynthesis
of Nanoparticles by Combinatorial Screening in Scanning Electrochemical
Cell Microscopy. Nanoscale.

[154] Wang Y., Garcia-Carrillo R., Ren H. (2025). Kinetics and Dynamics
of Atomic-Layer Dissolution on Low-Defect Ag. Chem. Sci..

[155] Tetteh E. B., Banko L., Krysiak O. A., Löffler T., Xiao B., Varhade S., Schumacher S., Savan A., Andronescu C., Ludwig A., Schuhmann W. (2022). Zooming-in
– Visualization of Active Site Heterogeneity in High Entropy
Alloy Electrocatalysts Using Scanning Electrochemical Cell Microscopy. Electrochem. Sci. Adv..

[156] Tarnev T., Aiyappa H. B., Botz A., Erichsen T., Ernst A., Andronescu C., Schuhmann W. (2019). Scanning Electrochemical
Cell Microscopy Investigation of Single ZIF-Derived Particles as Electrocatalysts
for Oxygen in Alkaline Media. Angew. Chem.,
Int. Ed..

[157] Wang Y., Gordon E., Ren H. (2019). Mapping the Nucleation
of H_2_ Bubbles on Polycrystalline Pt via Scanning Electrochemical
Cell Microscopy. J. Phys. Chem. Lett..

[158] Hengsteler J., Mandal B., Van Nisselroy C., Lau G. P. S., Schlotter T., Zambelli T., Momotenko D. (2021). Bringing Electrochemical
Three-Dimensional Printing to the Nanoscale. Nano Lett..

[159] Klausen L. H., Fuhs T., Dong M. (2016). Mapping Surface Charge
Density of Lipid Bilayers by Quantitative Surface Conductivity Microscopy. Nat. Commun..

[160] Fuhs T., Klausen L. H., Sønderskov S. M., Han X., Dong M. (2018). Direct Measurement of Surface Charge Distribution in
Phase Separating Supported Lipid Bilayers. Nanoscale.

[161] Online Open Access Programme and Meeting Document Understanding Open Science; 2022. 10.54677/UTCD9302.

