# Supplementary material for: A Look inside a Flexible Open-Source Scanning Electrochemical Probe Microscope
Source: ACS Electrochem. 2025 Dec 4;2(1):78–91. doi: 10.1021/acselectrochem.5c00354 (PMC12766686; doi:10.1021/acselectrochem.5c00354)
Supplement: Supplementary file 1 [file ec5c00354_si_001.pdf]

Supporting Information for:

A Look inside a Flexible Open-Source Scanning Electrochemical Probe Microscope

Kim McKelvey,[*,†] Martin Andrew Edwards,[*,‡] Minkyung Kang,[*,§,||] Marc Brunet Cabré,[⊥] Nicholas B. Jones,[‡] and Patrick R. Unwin[*,§]

[†]MacDiarmid Institute for Advanced Materials and Nanotechnology, School of Chemical and Physical Sciences, Victoria University of Wellington, Wellington 6012, New Zealand

[‡]Department of Chemistry and Biochemistry, University of Arkansas, Fayetteville, Arkansas 72701, United States

[§]Department of Chemistry, University of Warwick, Coventry CV4 7AL, United Kingdom

[||]School of Chemistry, University of Sydney, Camperdown, NSW 2006, Australia

[⊥]School of Chemistry, Trinity College Dublin, Dublin 2, Ireland

*kim.mckelvey@vuw.ac.nz

*maedw@uark.edu

*minkyung.kang@sydney.edu.au

*P.R.Unwin@warwick.ac.uk

Table of Contents

S1: Parts list for SEPM S2

S2: Custom-fabricated components S6

S3: Scheme of the overall electrical connections for SEPM. S15

S4: Description of the data stored, feedback types, and waypoint options. S17

S5: LabVIEW program “Scan hopping.vi” S21

S6: Data processing scripts for “Scan hopping.vi” S26

S7: Access to software and designs S28

S8: Additional experimental details for examples 1 and 2 from the main text S29

S9: References S30

S1: Parts list for SEPM

The flexibility of the software described in this work allows SEPMs to be constructed from components supplied by a wide range of manufacturers, without requiring reprogramming or other labor-intensive modifications. Below is a list of parts that can be used to build a general-purpose SEPM, alongside suppliers for the parts. This list of suppliers and models is not exhaustive but includes components that have been used and tested as part of EC-SPMs in the authors' laboratories. Where custom-fabricated parts are required, they are listed here and discussed in more detail in Section S2.

Physical Components

(i) Instrument Control and Data Acquisition:

1 × FPGA-based data acquisition (DAQ) card. The card should be capable of running the control software written in LabVIEW. E.g., PCIe-7852R or USB-7855R, both from NI (formerly National Instruments).

1 × computer. The computer is used to control the scanned probe microscope and acquire data. The computer should have appropriate slots/connectors to host the FPGA-based DAQ card.

1 × connector/adapter allowing the voltage inputs/outputs of the FPGA-based DAQ card to connect to the various electronic components described below. The choice of this component will depend on the data acquisition card chosen. For an internal data acquisition card, e.g., the PCIe-7852R, a cable (SH68M-68M-EPM; NI) and a terminal block (SCB-68A) can perform this role. Alternatively, a homemade breakout box, which connects the inputs/outputs of the data acquisition card to BNC connectors, can be used in this role. The internal contents of the breakout box may be a screw-terminal connector block, a USB-connected FPGA-based DAQ card, or a cable coming from a data acquisition card situated in the computer.

Cables (multiple): Cabling provides electronic connections between the data acquisition card and other hardware components. The connectors should match those on the breakout box and the components used, with BNC connectors being a common flexible solution to this task.

(ii) Vibrational and electrical isolation:

1 × vibration isolation platform (examples are a S-2000 from Newport and the 25BM-8 platform from MinusK)

1× Faraday cage[1] – this may be homebuilt or commercial. At a minimum, this should contain the elements through which low currents are flowing (wires, (pre-amplifier), electrochemical cell), although frequently the entire scanning portion of the instrument is enclosed within the cage. Piezoelectric controllers and other large electronic components (see below) are placed outside of the faraday cage on separate supports. To aid in damping vibrations, it is recommended to line the faraday cage with acoustic foam (e.g., Foam Acoustic Insulation 408-8228 from RS Components). For the highest resolution measurements where the highest stability is required, thermal drift (changes in the position of components due to changes in temperature and different thermal

expansion coefficients) can be detrimental. In these cases, it is recommended that the mitigation procedures detailed in reference 2 are followed.

(iii) 3-D Positioning System:

The fine positioning portion should consist of either one z piezo and one xy piezo or one xyz piezo with appropriate amplifiers. A key requirement of the amplifier is that it can use an analog signal as an input to control the position. It is preferable that the piezo amplifier can report the position of the piezos, also via an analog signal, but that is not necessary for the software to function. As amplifiers must be matched to piezos, we recommend discussing with manufacturers to obtain an appropriate amplifier.

z piezo – P-753.3CD with amplifier E-665.CR (Physik Instrumente)

xy piezo – E-621.2CD with amplifier 2× E-625.C0 (Physik Instrumente)

or

xyz piezo – Nanocube™ P-611.3S with E-644 Nanocube™ piezo controller (Physik Instrumente)

1 × 3-axis micropositioner – e.g., the M-461-XYZ-M (Newport)

3 × micrometers or adjustment screws – e.g., 3× SM-25 (Newport) or (optional) 1 × stepper motor and controller, as a replacement for the z-axis micrometer. E.g., Picomotor™ Actuator (model 8302) with driver module (model 8703) and power supply 8704NF (all from Newport)

The positioning systems listed above are representative examples. We have successfully built SEPMs using piezoelectric positioners from a wide variety of manufacturers, including, but not limited to: Physik Instrumente, Piezosystem Jena, Mad City Labs, and nPoint. The builder is encouraged to consider the range of the piezos, with respect to: 1) the dimensions and roughness of the samples they plan to image and the type of experiments they plan to perform, e.g., an SECM approach curve with a microelectrode would require a larger z-range than SECCM imaging of 2-D materials, and 2) the resolution and temporal response of the piezoelectric positioners.

(iii) Current/Voltage Amplifier:

Current/voltage amplifiers capable of recording sufficiently small currents (typically nA-pA) or voltages (µV to V) and operating with a sufficient number of channels for the technique to be performed are required. These should be interfaced with the FPGA-based DAQ card through analog signals. Numerous examples of such amplifiers are commercially available and have been successfully integrated in SEPM-controlled instruments. These include instruments from Dagan (Chem-Clamp), Molecular Devices (Axon Instruments), and patch clamp amplifiers from Warner Instrument Corporation, Femto (DDPCA-300, DLPCA-200), NPI Electronic (VA-10M), HEKA (EPC 10 USB), as well as conventional potentiostats/galvanostats from CH Instruments and Gamry. Warwick Electrochemistry & Interfaces Group at the University of Warwick operates SEPM techniques with custom-built instruments, developed by Dr Alex W. Colburn (Colburn Electronics, Coventry, UK).

(iv) Probes

All SEPM measurements require probes, which are typically some form of nano- (micro-) electrode or pipette. Their fabrication is beyond the scope of this article, but interested readers are directed to references 3–6. Several commercial vendors exist for electrochemical probes; these include Park Systems (nanopipettes) and CH instruments (microelectrodes).

(v) Miscellaneous (Interface plates, sample and electrode holders, bolts, …)

Connecting the mechanical components (e.g., the piezos and micropositioners) requires interface plates that are machined with a hole pattern matching the two components to be interfaced, which are then bolted together using bolts with the appropriate thread. The probe holder, which mounts to the piezo, must have an appropriate hole pattern and a receiver for the probe being used. Moreover, samples come in many shapes and sizes, and no one sample holder suits all samples. For these components, there is no one-size-fits-all solution, and the components must be custom-fabricated. Designs for these custom components are discussed in section S2 of the supporting information. All components for the positioning system and signal amplifier head stages are physically mounted either directly onto an optical table or a breadboard (Figure 3, main text; Figure S2, supporting information).

Software

(vi) The software that controls the instrument is written in LabVIEW 2020. To run the software requires a license to LabVIEW that includes the FPGA module (this module is included in the 'Full' and 'Professional' versions and may be included in some educational licenses). Licenses to more recent versions of LabVIEW also appear to run the software, although not all versions have been thoroughly tested. Users may also need to download drivers compatible with the FPGA-based data acquisition card they selected.

Optional physical components

(vii) Optics for Alignment

One (or a pair of orthogonally mounted, optical camera(s)) (e.g., PL-B776 from Pixelink) equipped with high magnification telecentric lens(es) (e.g., 3.0X InfiniFlex HD Compact Lens from Edmund Optics) and/or an inverted microscope (Axiovert 40 CFL from Zeiss) can be aligned with the sample, to facilitate positioning the probe in the vicinity of the area of interest.

(viii) Lock-in Amplifier and Voltage Adder

For measurements employing oscillating signals, e.g., oscillating the probe height or potential, a lock-in amplifier (such as a SR830 from Stanford Research Systems) can be added into the SEPM system. The lock-in amplifier generates a sinusoidal signal (zero offset). A measured signal, typically the current, is fed into the lock-in amplifier, which measures a component (amplitude and or phase) of the signal that oscillates at the same frequency. The extracted amplitude and phase are then fed into the data acquisition card (see Figure S8 for connections).

Frequently, when an oscillating signal is used, we desire to apply the oscillations around a non-zero value, e.g., oscillations about the tip's average position. While certain models of components (e.g., piezo controllers, current/voltage amplifiers) might have options to add together an external (in this case, AC) input to a second (DC) signal, when this is not possible, the two signals should be added together prior to passing to the component. This is achieved by a voltage adder or summing amplifier (see Figure S8 and discussion for an example of where a voltage adder is used). Many simple circuit designs exist for summing amplifiers, allowing users to build their own. The Warwick group uses a custom-built summing amplifier (Colburn Electronics, Coventry, UK). Inexpensive commercial models are available, but the authors have not tested any. An important consideration of any such amplifier is the frequency response, which must adequately pass the sinusoidal signal with minimal distortion.

S2: Custom-fabricated components

As described in section S1(v), building a SEPM requires fabrication of several custom components. For example, mounting the probe to the piezo requires a probe holder that is suited to both the size of the probe and to the threaded holes in the piezo, while attaching the piezo to the micropositioner stage requires an interface plate that is drilled with holes that match the two components. Designing and fabricating these components might appear daunting for those building their first SEPM, particularly if they lack experience with fabrication techniques. To lower this barrier, our flexible open-source SEPM includes editable component designs and fabrication pathways, making it easier for anyone wanting to construct their own SEPM.

Below, we describe example designs for each of the custom components required for an SEPM. They were fabricated to function with a specific selection of stages and piezo positioners and are representative of a range of downloadable designs available. We then describe where editable designs for other component combinations can be downloaded. Finally, we provide routes to fabrication for those without access to the appropriate tools or expertise to fabricate the components themselves.

Designs

To begin the process of fabricating the custom parts, the specific components must be chosen following the guidance provided in section S1. Three important parts that need to be decided on before the custom parts can be designed are: the piezo actuator, the linear stage, and the optical breadboard or base for the microscope. In each case, these choices affect the pattern of holes that allow the components to be attached together. Below we provide typical examples of these components that are compatible with a TRITOR 100 piezo actuator (Piezo System Jena) a 9067-XYZ-M 3-axis linear stage (Newport) and M-SA2-22 optical breadboard from Newport.

Probe holder.

The probe holder connects the piezo (z or xyz depending on whether the xy and z axes are separated or combined in single actuator, respectively) to the probe, which is typically a glass cylinder ranging from 1-2 mm. Figure S1 shows a probe holder compatible with the components listed above. It consists of a flat plate with holes to screw it to the piezo and an arm that extends out over the sample, and that contains a vertical groove which supports the probe.

The specifications for the piezo bolt holes (and for other components described in this section) can typically be found on the manufacturer's data sheet/engineering drawings. E.g., for the TRITOR 100, these are 4× rectangularly spaced M3 holes with center-to-center spacings of 32 mm (vertical) and 20 mm (horizontal). We designed the holder to support a nanopipette with a diameter of 1 mm or larger.

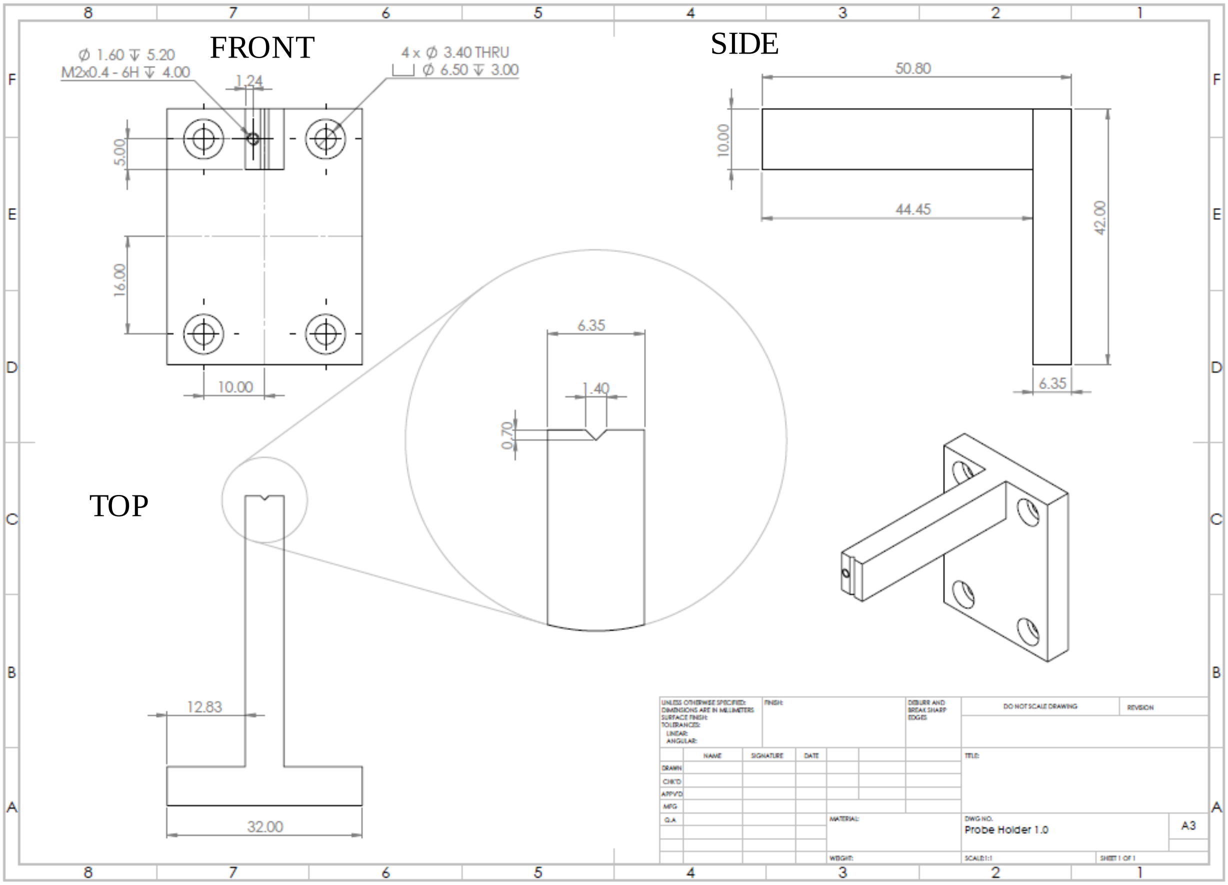

Figure S1. Top: engineering drawings for a probe holder for an SEPM to mount a 1 mm diameter probe to a TRITOR 100 piezo. Bottom: 3D-rendering of the probe holder.

To support the probe a vertically aligned v-channel cutout was placed at the end of the holder (see top view and zoom in circle in Figure S1). The depth of the cutout (0.7 mm) was chosen to be slightly smaller than the smallest diameter of the probe to be secured (1 mm) so the thumbscrew and nylon washer could apply ample pressure to secure the probe (see Figure S2 for the manufactured holder including thumbscrew and washer).

Figure S1(top) shows an engineering drawing of the probe holder. The holes are described with callouts. For this holder there are two types of hole: 1) the hole for the thumbscrew calls out an M2 bolt and indicates it should be threaded for such, 2) bolts that will attach to the piezo are labelled “3.40 THRU ALL.” This hole is for a M3 bolt to pass through and indicates it in that it has a clearance for 3.40 mm. Note: This hole does not have a thread tapped in it, as the bolt screws into the tapped hole in the piezo. All units are indicated as ‘millimeters’ in the table inserted in the engineering drawing (bottom right).

The fabricated probe holder, including thumbscrew, nylon washer, and probe, is shown in Figure S2. Showing clearly how the washer applies pressure to the probe (a glass nanopipette).

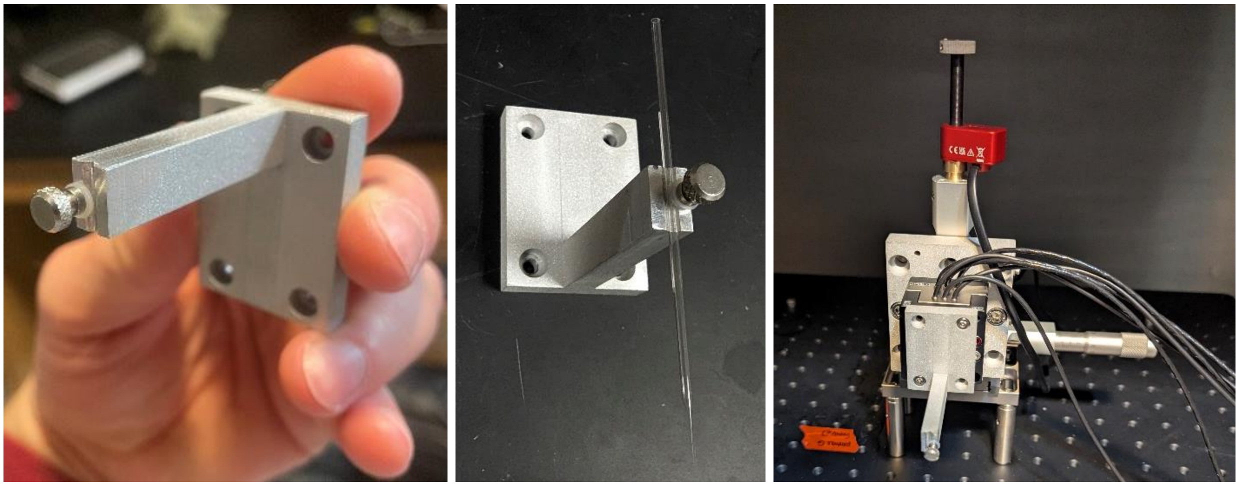

Figure S2. Probe holder for an SEPM to mount a 1 mm diameter probe to a TRITOR 100 piezo. Left) Part machined from Al, including M2 thumbscrew and nylon washer for securing probe. Center) Unfilled nanopipette probe secured in holder. Right) Probe holder mounted to the piezo and linear stages.

Piezo-to-linear-stage interface plate

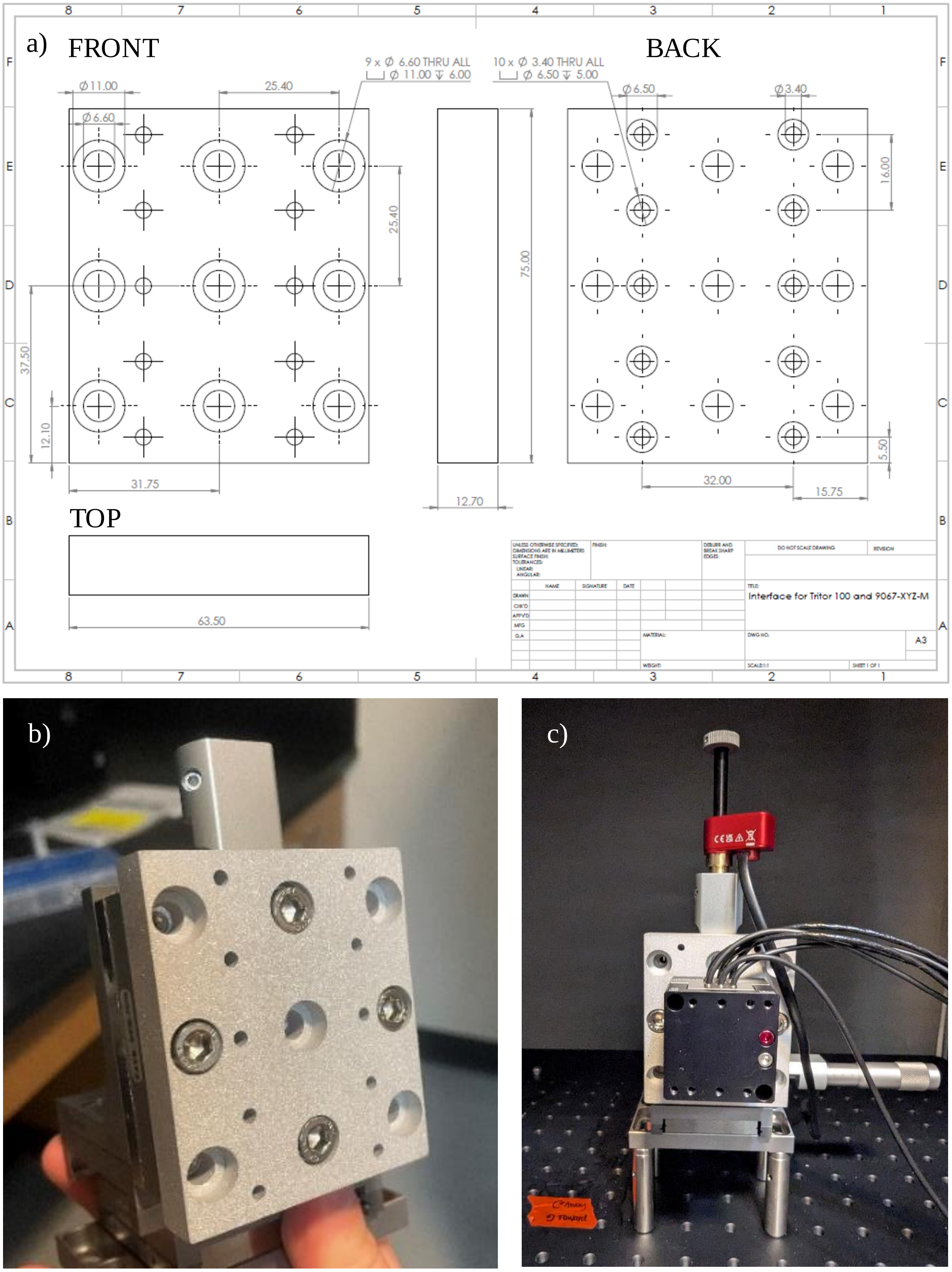

Figure S3. Interface plate for attaching TRITOR 100 piezo actuator to a 9067-XYZ-M linear stage. a) Engineering drawings. b) Part machined from Al attached to linear stage by 4 M6 bolts. c) Plate with piezo mounted.

An interface plate is used to attach the piezo (z or xyz) to the linear stage. Figure S3 shows such a plate for attaching a TRITOR 100 piezo actuator (Piezo System Jena) to the 9067-XYZ-M XYZ linear stage (Newport). For its design, attention must be paid both to the hole patterns and bolt size (see above) and the order of assembly. Attaching to one component might block some holes intended for mounting to the other. This can be remedied by ensuring ample alternate holes on the plate following the hole pattern. E.g., in this design, there are five pairs of holes separated by 16 mm center-to-center vertically and 32 mm center-to-center horizontally, providing three possible placements for the piezo that has attachment points on a square with 32 mm center-to-center spacing. Having multiple attachment options also adds flexibility to the instrument. When measuring particularly high or low samples, the entire piezo and probe-holder assembly may be raised or lowered.

In this design, holes are countersunk to create space for the bolt head. This allows components to lie flush against each other. Although this requires a relatively thick metal plate, this weight is not moved by the piezos and therefore does not reduce the piezo's movement speed.

Sample holder and mount

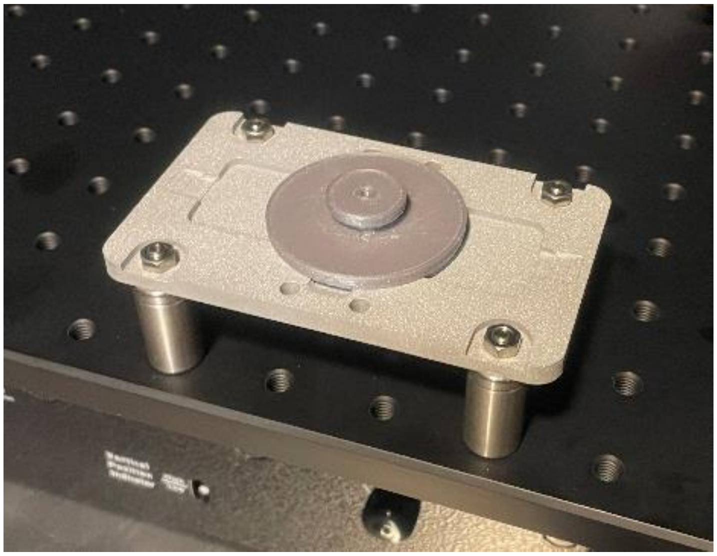

Figure S4. A sample holder capable of supporting a microscope slide or a ‘puck’ (see Figure S5 for engineering drawings). Part machined from Al, mounted to breadboard using optical posts. A ‘puck’ style sample support is mounted in the sample holder. Optical posts are supporting the sample holder above the breadboard.

Users may want to measure samples with a wide range of dimensions using a SEPM. Sample mounting options should support this range of samples and allow quick and easy sample switching. To achieve this, we use a two-part mounting system consisting of a fixed base mount that is attached to the breadboard (fixing to a solid base plate is also possible) with switchable sample pucks, an example of which is shown in Figure S4.

The base mount shown in Figure S4 is designed to hold samples mounted on nylon/PTFE pucks and glass slides. Engineering drawings for the base are provided in Figure S5. It is designed with a hole pattern matched to the grid of the threaded holes in the breadboard (M-SA2-22 optical breadboard, Newport). As shown in Figure S4, the sample base is mounted above the breadboard on optical posts, allowing for back access to deep samples, e.g., imaging of a cylinder macroelectrode to which a wire needs to be attached.

The notches in the base mount allow easy extraction of the sample puck/microscope slide. The four holes near the center of the holder allow for the mounting of sprung clips, which can be used to secure samples.

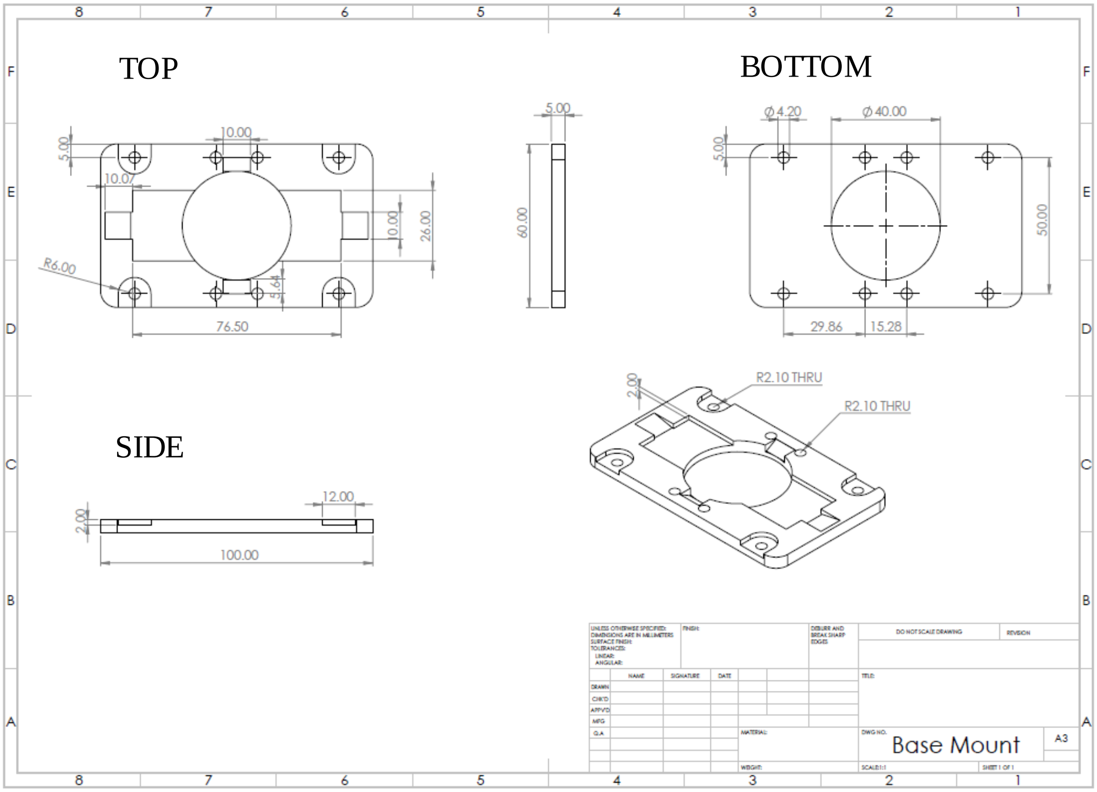

Figure S5. Engineering drawings. (bottom) of a sample holder capable of supporting a microscope slide or a ‘puck’.

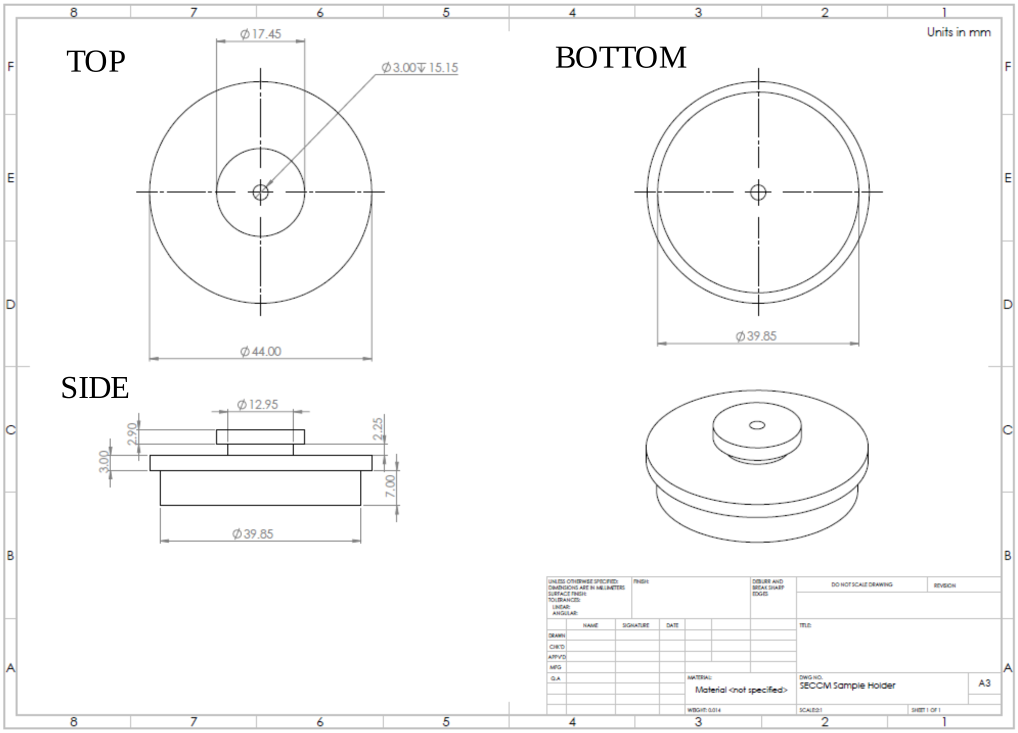

Figure S6. Engineering drawings of a puck sample holder.

The sample puck is machined to sit perfectly in the hole in the sample base by making it slightly smaller (39.85 mm vs 40 mm diameter in the examples shown in Figures S5/6). It should be fabricated from a material that is chemically inert to the solvents being used. The upper part of the sample holder will depend on the sample. In the example shown in Figure S6, a 17.45 mm diameter upper cylinder with a groove below it is designed to support a cylindrical glass tube and an O-ring. It would be suitable for a small sample that is to be immersed in a solution.

In our experience, it is wise to fabricate multiple puck sample holders, such that samples can be secured to them and quickly interchanged. Depending on the measurement being performed, it may be necessary to drill a hole through the back of the sample (e.g., when imaging a macroelectrode). For soft plastic pucks, these modifications can be performed quickly using hand tools.

Availability of designs

The specific details of the parts described above were chosen to be compatible with the specific components listed at the start of this section. While it would be impossible to provide designs for every different choice of component or sample, as an open-source instrument, we have made the above designs (both drawings and electronic files) available, alongside those for many of the most commonly used piezos and linear stages. We anticipate that as users design their own custom components, their designs will be uploaded, such that others may benefit from their efforts and expertise. Academic users are free to download and adapt existing designs, take inspiration from them, or design things from scratch.

The directory of parts is available as part of the open-source suite (see Supporting Information section S7 for access details) in a directory called ‘Parts’. Within the directory, the designs are hierarchically organized, starting with three folders representing the same three categories used above (“Interface Plates”, “Pipette Holders”, and “Sample Holder & Base Mount”). Within the “Interface Plates” folder, subfolders are named after the piezo actuators, which contain plans to mate the named piezo to various linear stage positioners. Similarly, in the ‘Pipette Holders’ folder, there are subfolders named after the piezo actuators and linear stages, each containing plans to attach a probe holder to each component.

Each plan is furnished with an engineering drawing (PDF), an opensource .stl file, and a Solidworks file. This is to extend compatibility and to provide a visual guide to each part. These designs are basic and can be modified for custom purposes.

Fabrication and materials

Once all the parts are designed, a good practice is to 3D print the parts and test-fit them before their fabrication from more expensive materials. 3D printers are generally accessible on many college campuses, public libraries, maker spaces, etc., and provide a physical test of the compatibility of parts and insight into how the part will function when installed. An example of a 3D-printed electrode holder prototype is shown in Figure S7, alongside the final Al version.

If a 3D printer, or the expertise to operate it, is not readily available, there are many web-based services such as Xometry,[7] Hubs,[8] Protolabs,[9] and many more that will 3D print (or machine) your parts and ship them to you based on 3D designs, such as those described above.

Once designs have been finalized, parts should be fabricated from appropriate materials. We find that Al represents a good balance between machinability, weight, durability, and chemical resistance. As such, it is the material we commonly use for the interface plate, electrode holder, and sample base. Machining of Al can be achieved using a CNC router. If users have access to a router and the experience or personnel to operate it, then these parts may be fabricated in-house. Alternatively, the web-based services listed above all have the capabilities for CNC milling and other fabrication processes out of a wide range of materials, including Al, and so offer a relatively inexpensive route to fabrication for those without the required resources or expertise.

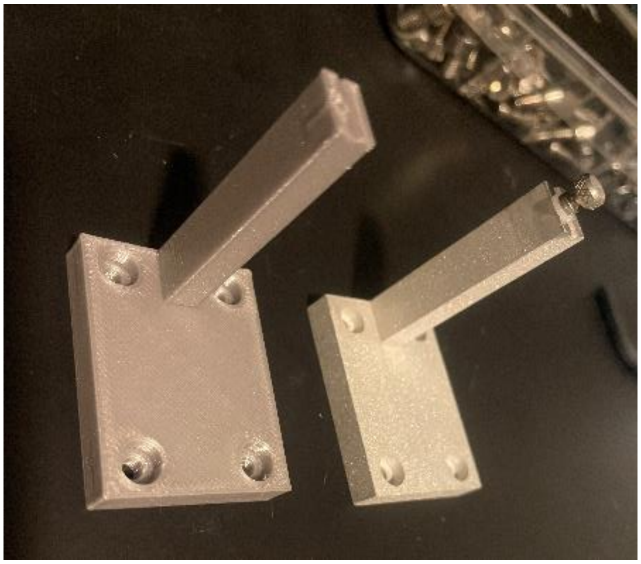

Figure S7. Electrode holder prototype fabricated by 3D printing (left) and final product CNC milled Al (right).

S3: Scheme of the overall electrical connections for SEPM.

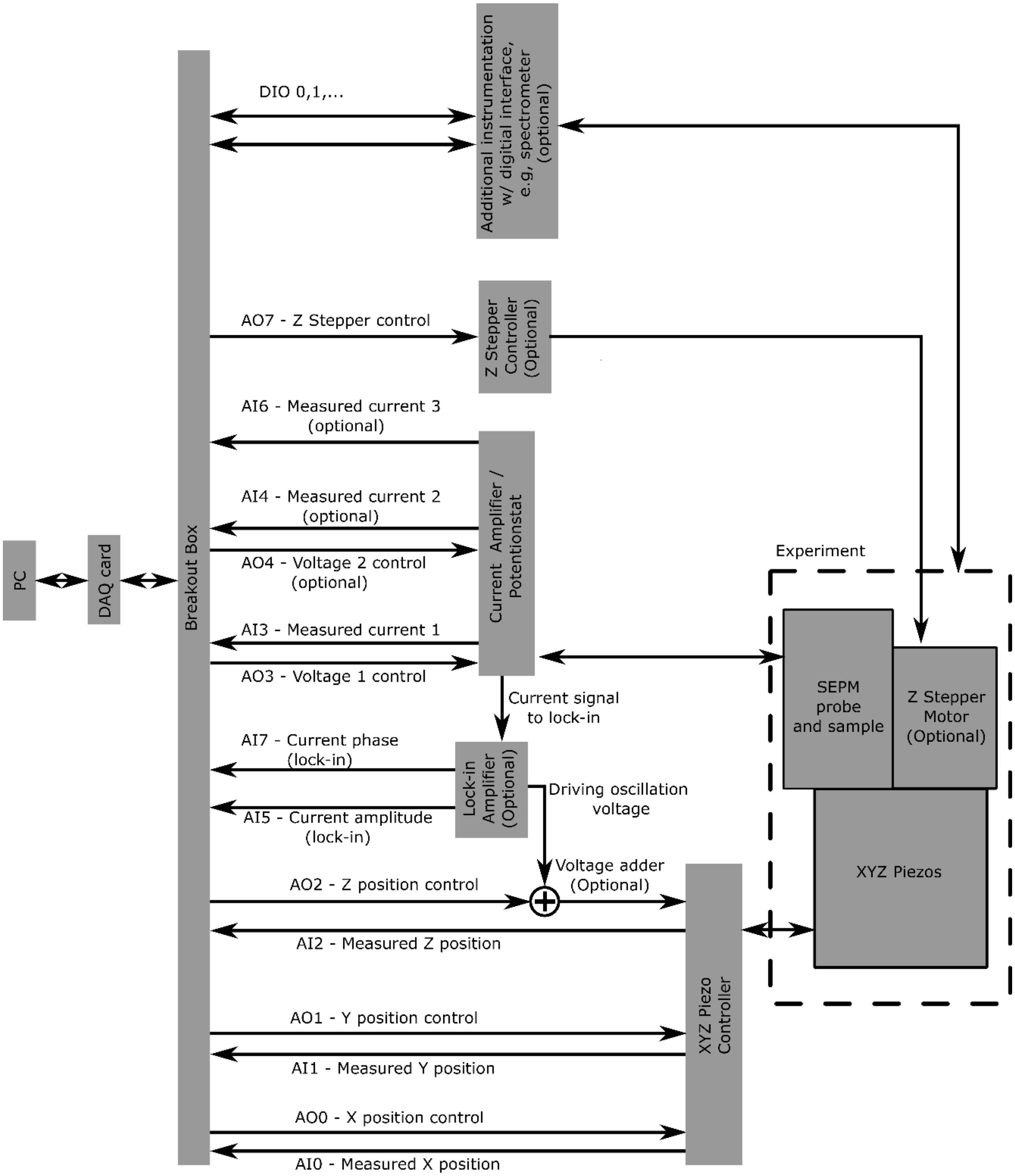

Figure S8. Schematic of components and connections in a SEPM. The direction of the arrows represents the direction of signal transmission; bidirectional arrows indicate information is passed in both directions. The labels AO (analog out), AI (analog in) and DIO (digital input or output) represent the default channels of the data acquisition (DAQ) card used to communicate the information.

Figure S8 shows the electrical connections in a typically configured SEPM instrument (e.g., for the measurements shown in Figure 6 and 7of the main text). The directionality of the arrows indicates where a signal is being transmitted to. As described in *Instrument Control and Data Acquisition* in the main text, these signals are voltages that are linearly related to the various physical quantities they represent. The diagram shows the option where the X, Y, and Z piezos are controlled by a single unit; however, they may also be sent to separate controllers.

The setup shown in the figure contains many optional components that are discussed in section S1 and the main text, which are not required for the instrument to function. E.g., voltage 2 and current 2 might be used in SECM-SICM, dual-electrode SECM, or dual-barrel SECCM (see Figure 1 in the main text for details). However, if optional control signals (Voltage 2') are not being used to control the listed quantity, they can be used to control other analog devices. Similarly, optional inputs (Measured Current 2', 'Measured Current 3') can be used to record additional analog signals, which can then be converted into physical quantities.

In the setup shown, a driving oscillation is added to the Z position, meaning that the probe's vertical position is oscillated. The current is being fed into the lock-in amplifier so that oscillating components can be extracted from it. This is a typical setup for dual-barrel SECCM[10] and tip-position modulation (TPM) SECM[11], and is sometimes used in SICM.[12]

If the piezo controller lacks a 'position out' option, it is recommended to connect the corresponding analog out cable directly to the analog input. However, when this is done, the 'measured position' as reported in the software is instead the commanded position.

S4: Description of the data stored, feedback types, and waypoint options.

During an experiment, data is streamed continuously to the hard disk in a NI Technical Data Management Streaming (TDMS) file format (see ni.com for a full description[13]) to a temporary file, as defined in *Settings.vi*. Thirteen separate channels (listed below in Table S1) are streamed to disk at a data rate of one set of measurements per period, δt_{data}. The data collection settings define the period through N, the number of samples to average, and δt_{sample}, the sampling period (both found in *Settings.vi*), by $\delta t_{data} = \delta t_{sample} \times (N+1)$ (the extra period is used to transfer the data).

Data Channel Name	Units	Description	Breakout Box Connector
X	µm	Measured X Piezo Position	AI-0
Y	µm	Measured Y Piezo Position	AI-1
Z	µm	Measured Z Piezo Position	AI-2
V1	volts	Voltage 1	AO-3
V2	volts	Voltage 2	AO-4
Current1	amperes	Current 1	AI-3
Current2	amperes	Current 2	AI-4
Current3	amperes	Current 3	AI-6
Feedback Type	integer	Feedback type for z-piezo control. See Table S2 details.	
Line Number	integer	Indicates the waypoint number being approached (starts = 1 and increments by 1 as each waypoint is reached)	
Lock-in Amplitude	amperes[1]	Oscillation amplitude (rms/peak-to-peak depending on the lock-in model)	AI-5
Lock-in phase	degrees	Oscillation phase vs the reference phase (from lock-in amplifier).	AI-7
δt_{data}	seconds	Time between data point and the preceding data point (see above for definition)	

Table S1. List of data channels that are recorded during measurements with the software.

[1] This presupposes that the current is being fed into the lock-in amplifier; a common setup that is shown in Figure S8. If other inputs are fed to the lock-in units will be different.

On completion of an experiment, a dialog is opened with the option for the user to convert data saved in the .tdms file format into a tab-separated values (.tsv) text file, which is human-readable and can be universally imported into any data processing software. A copy of the temporary .tdms file is also made, with the file name as defined in the dialog. Note the tdms data file format can be directly loaded in many data processing software, either directly or through additional packages, e.g., MATLAB (tdmsread command[14]) Microsoft Excel (with an add-in[15]), and Origin (OriginLab corp.) (Data > Import from File > NI TDM (TDM, TDMS)), or Python (npTDMS package[16]). See Supporting Information section S6, for an example of importing and processing data using MATLAB.

The *waypoint number* type is an integer value that starts at 1 and increments by 1 with every new waypoint. It is particularly useful for partitioning the data into sections, such as the forward and reverse parts of a CV or the approach and retract parts of the hopping mode scan.

Upon completion of an experiment, a '.set' text file is also generated, in which all the settings of the task are recorded in a human-readable text file format. The format of the data saved in the .tsv file is also recorded at the end of the .set file.

In summary, three files are generated for every task, a .tdms file (and an associated .tdms_index file) containing direct streams of all 13 channels of data, a .tsv file with selected channels converted to a text file format, and a .set file containing the settings used for the task.

Waypoint options

In addition to the options listed in Table 1 (main text), the full definition of each waypoint includes many more options. At the time of writing, the options are: the positions (X, Y & Z in μm) voltages (V1 & V2 in V) and rate of change for these quantities (μm/s or V/s); feedback type (see Table S2 below); whether to change or hold each of the quantities during the particular waypoint (boolean) and whether to jump or sweep each potential (boolean); hold (boolean) and hold time (μs), which when hold is 'true', causes the waypoint to last for at least the duration specified by hold time; whether to move the x/y/z picomotors (boolean) and which direction (boolean), NB: the distance of the move is controlled by the duration of the waypoint, as controlled by the hold time; how often the z-position feedback loop updates the position (μs).

These parameters are described in more detail in the developer's guide, which is downloaded from the repository (see section S8). This document is updated as new feedback types and waypoint options are added to the code.

Feedback Types

As described in the main text, the way in which the z-position is updated is a key defining parameter in SEPM experiments, e.g., see constant height vs constant distance vs hopping scanning (Figure 2, main text). The full list of feedback types is extensive and is listed in Table S2. The final column provides references to examples of experiments using the listed feedback method and provides further details of when a particular type of feedback might be useful.

Feedback Type	Description	Example Applications and references
0	No feedback (default).	Move probe/sample to a initial position of the scan. Constant height scanning.
1	Pause when set point(s) are obtained.	Approach the probe to within a specified distance of the surface (e.g., set the tip-sample separation in SECM or SICM) and wait for the experimenter to intervene.
2	Move to the next waypoint once a threshold value is observed	Used in hopping mode imaging and modified approach programs that perform actions after approaching a probe to a certain distance from the surface.[17–21]
3	Move the *z* position by an amount relative to the current *z* position.	Retract the probe away from the surface in hopping mode imaging or for SICM reaction mapping (see Figure 7 in the main text and associated discussion). [17–21]
4	Maintain a set point using a proportional controller to adjust the *z*-position.	Used in constant distance imaging.[22–24]
5	Maintain a set point using a proportional controller to adjust the *z*-position, while storing the height. The recorded height can be used, with Feedback Type 6, to retrace the topography of the surface.	Used for storing the *z*-position during constant distance. [25,26]
6	Retrace the height stored by Feedback Type 5. Assumes that the *y* position and the number of *x* points for the line are identical to those for Type 5.	Retrace the *z*-position of the probe stored from the previous scan.[25,26]
7	Maintain set point using a proportional controller to adjust the z-position and pause movement when a second independent set point is achieved.	N/A
8	Measure the average current value at the start of the movement towards the waypoint (typically in bulk solution). Move to the next waypoint based on a threshold difference from the initial measurement.	Maintain stable tip-sample separation regardless of marginal drift in "bulk" signal, used in hopping mode imaging, especially over extended periods.[27–31]
9	During probe movement, keep a running average of the current, and move to the next	N/A

	waypoint based on a threshold difference from this running average.	
10	Maintain a set point using a proportional controller to adjust the *z*-position, but only for positive error values.	N/A
11	Maintain a set point using a proportional controller to adjust the *z*-position, but only for a limited time.	Maintain a fixed *z*-position while measuring at a point for a defined time.[32]
12	Move the *z* position by an amount relative to the current *z* position. A different distance from 3 and 13.	Used to develop different scanning patterns.[32–34]
13	Move the z position by an amount relative to the current *z* position. A different distance from 3 and 12.	Used to develop different scanning patterns.[32]
14	Measure the average current value at the start of movement (typically in bulk solution), then move to the next waypoint once a percentage deviation from this initial value is observed.	Modified Feedback Type 8 for stable SICM hopping mode imaging.[33,35]

Table S2: Feedback types for controlling the vertical movement of the probe. Reference column gives literature examples of using the feedback described.

The list of feedback types is comprehensive at the time of writing. However, new feedback options may be implemented and pushed to users through the repository. The most up-to-date list of feedback types can be found in the developer's guide, which is available for download from the repository (see section S8).

S5: LabVIEW program "Scan hopping.vi"

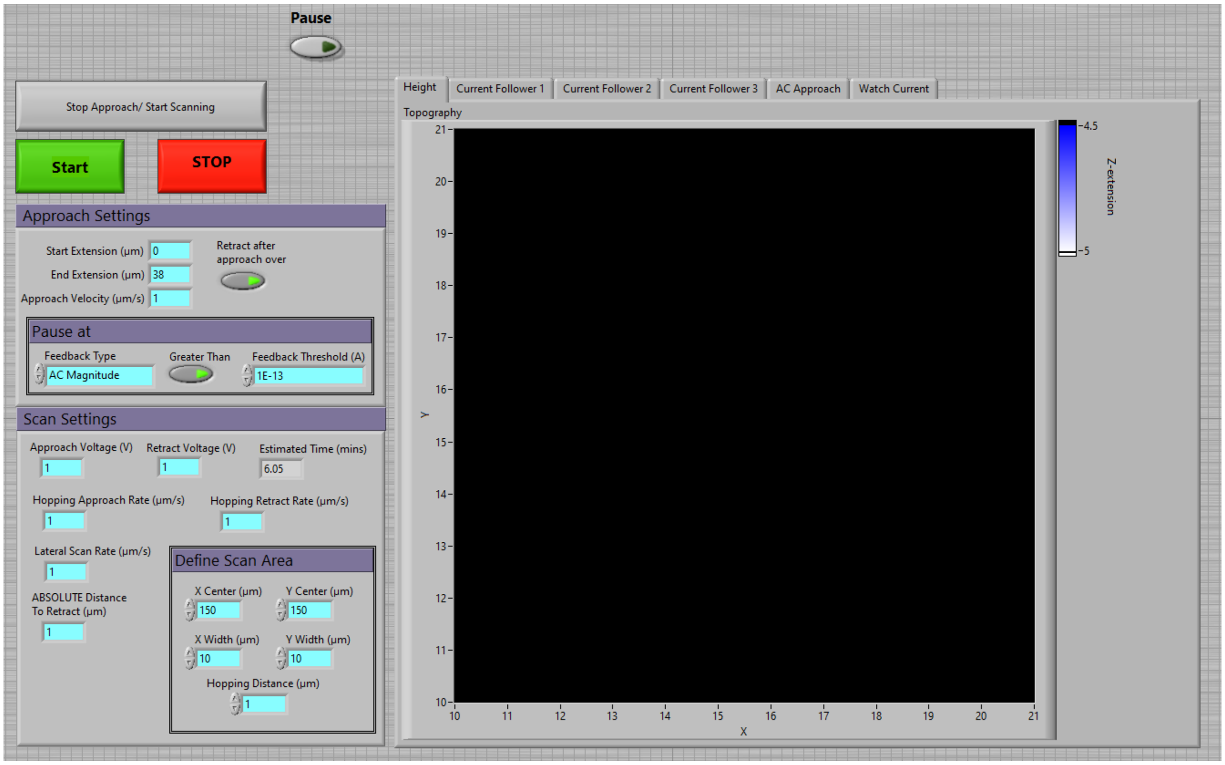

Figure S9. Front panel for "scan hopping.vi", with settings chosen to perform a hopping scan at an 11×11 array of points. The parameter values on the left-hand side determine the region of the scan, the movement of the probe, and the threshold used to determine proximity to the surface. The tabs on the right-hand side of the figure will present the data in real time as the experiment progresses.

Figure S9 shows the front panel of the 'Scan Hopping.vi', a program that was used for the experiment described as 'Example 1' in the main text (for the back panel, see Figure S10). The parameters are grouped together based on their function. The "Approach Settings" parameters describe the initial approach to the surface, in this case using the full range of vertical movement (0-38 µm) and the speed of approach of 1 µm/s. "Pause at" determines which quantity is monitored to determine proximity to the surface, and at what value the threshold is met and the surface is deemed to be located. These z values are used to generate topographic images, such as that shown in Figure 6C, main text. The "Scan Setting" parameters include defining the dimensions of the scan ("Define Scan Area"), the speed of the probe movement, and the voltage that is applied to the probe/substrate. The graphs, which appear in the tabs on the right-hand side and the parameters discussed above, all appear as icons in the block diagram, which is shown in Figure S10.

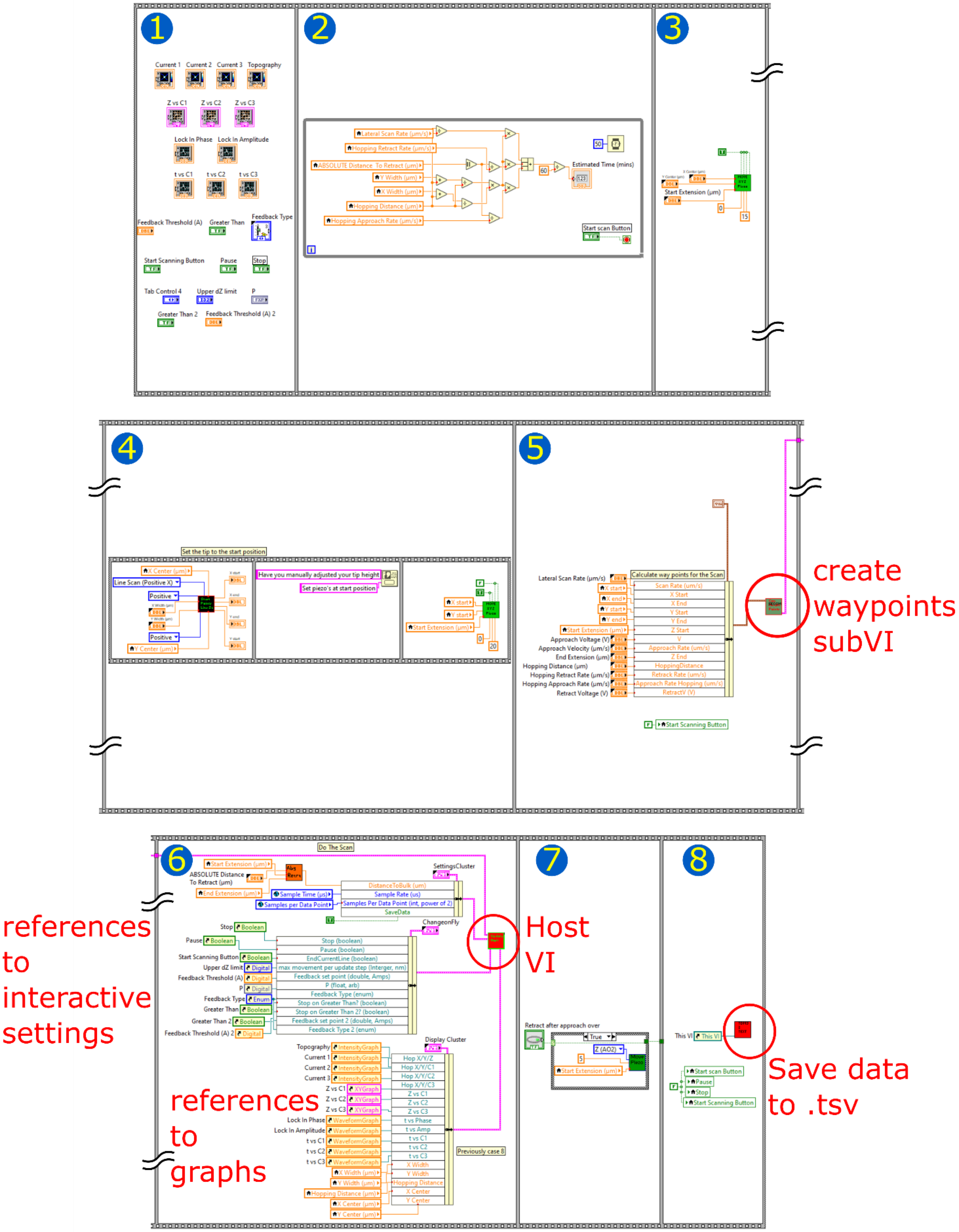

Figure S10. Block diagram for "Scan_hopping.vi" code. Numbers and text annotation have been added to aid discussion.

The back panel of the Scan Hopping.vi experiment (Figure S10) is organized into a sequence of steps (1-8). The purpose of each step is:

Step 1: Define all local variables. This step in the sequence exists to arrange all the local variables in one place and make them easy to locate.

Step 2: Calculate the estimated scan time from the values on the front panel. This value continually updates with the user changes values until they click the 'start scan' button.

Step 3: Move the probe to the lateral (*x*,*y*) center of the scan area. This step allows the user to scan over a feature of interest on the sample surface.

Step 4: Calculate the start and end points of the scan from the center/range of the scan (this is done by a subVI). It displays a dialog box, which gives the user an opportunity to manually move the probe's position laterally to the (*x*,*y*) start position of the scan.

Step 5: Calculate the waypoints needed to perform the scan based on the front panel values. This task is performed by a separate subVI, which is shown in Figure S11.

Step 6: Perform the scan. This is initiated by calling the Host.vi with the waypoints needed to conduct the experiment, all appropriate scan settings, and references to input/output elements that may be modified during the experiment, such as the graphs where data will be plotted, the stop and pause buttons, and feedback settings.

Step 7: Optionally, retract the probe after the end of the experiment. In this experiment, retracting the probe from the surface occurs separately after the experiment has been completed, as opposed to as part of the waypoints. This structure is used so that if the user aborts the experiment (by pressing the 'Stop' button), the probe is still retracted from the sample surface.

8: Convert data from TDMS format to TSV format. Once the experiment is complete, the user has the option to define the file names and convert some/all of the data from the TDMS file format to a plain text tab-separated variables (TSV) file format (the TDMS file containing all the data is always stored). Values of buttons are reset in preparation for the next running of the VI.

Figure S11 shows the subVI for generating waypoints for the 'hopping scan.vi' (see Step 5 above). This component of the software is best viewed within LabVIEW. It loops over each of the points in the scan in a manner described in Figure 4 of the main text.

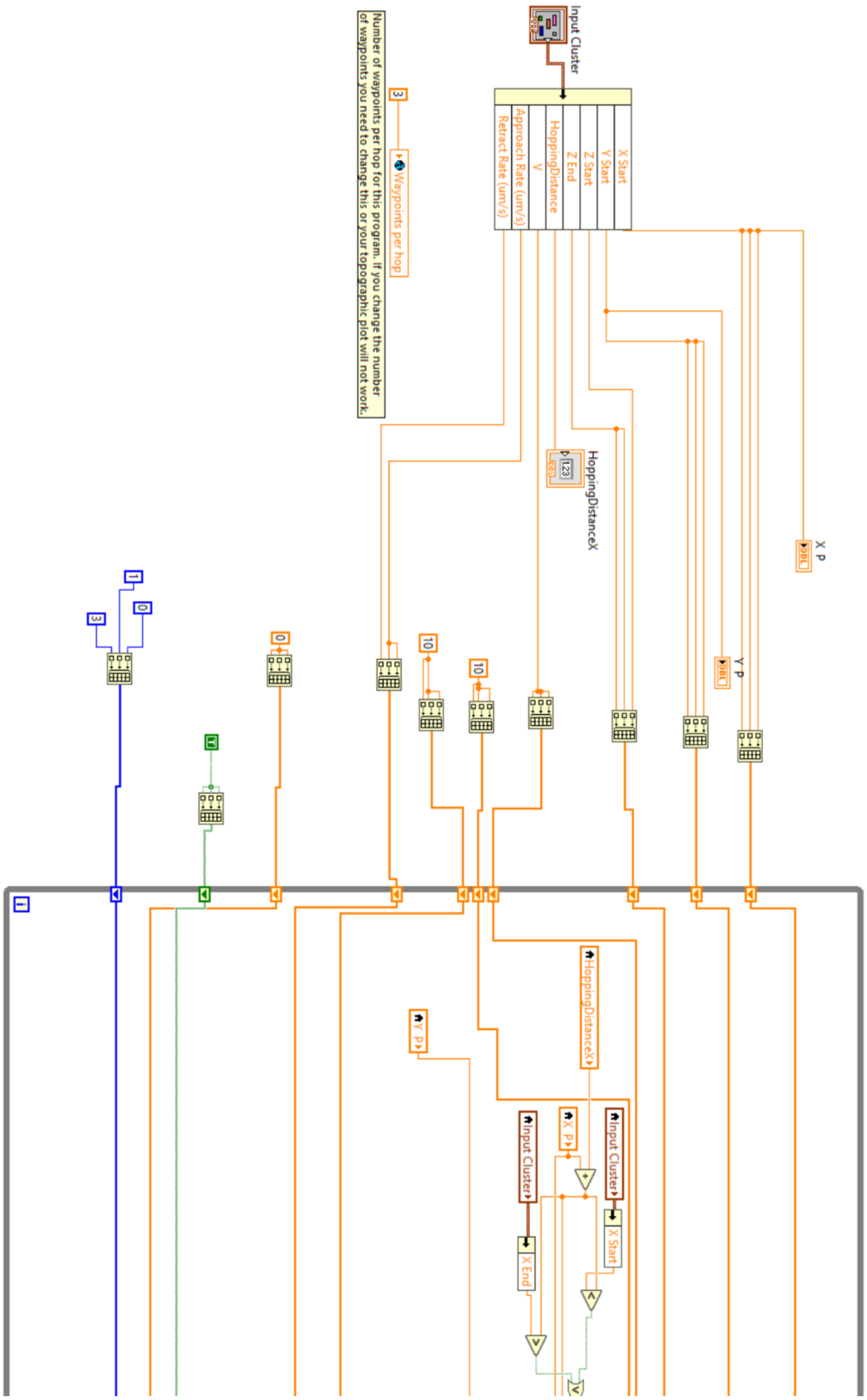
Input Cluster
X Start
Y Start
Z Start
Z End
HoppingDistance
V
Approach Rate (um/s)
Retract Rate (um/s)
Waypoints per hop
Number of waypoints per hop for this program. If you change the number of waypoints you need to change this or your topographic plot will not work.
HoppingDistanceX
X P
Y P
X End

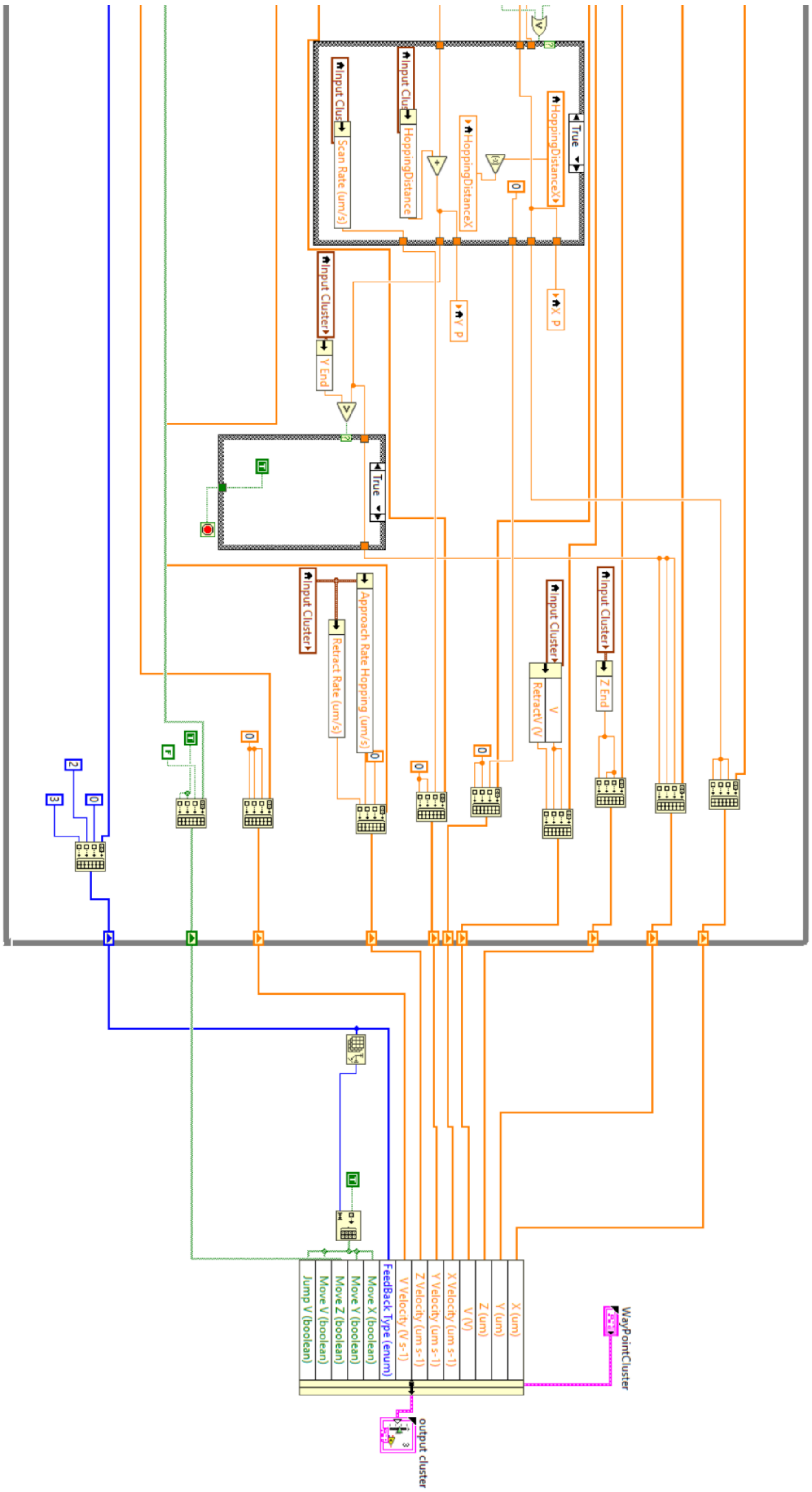

Figure S11. Block diagram of the subVI used to generate the waypoints for scan hopping.vi.

S6: Data processing scripts for "Scan hopping.vi"

Given the flexible nature of the instrument and the infinite number of possible experiments and types of data analysis, there is no single way to analyze data from the flexible SEPM software described in this work. However, many of the steps in processing and analyzing data remain conserved through a wide range of experiments (import data, extract experimental settings from .set file, extract specific data points from expt., plot data, store extracted data). The script below performs these steps for an SECCM hopping scan experiment (such as shown in Example 2 of the main text), generating a plot of the topography from the last point of each approach to the electrode surface. The script is written in the MATLAB programming language, but a similar structure could be used for other procedural programming languages.

The data file required to run this script, `ScanHopping.tsv` (available from https://doi.org/10.5061/dryad.9p8cz8wx3) should be placed in the same directory as the script. This script may be used as the basis for analysis of different experiments and more advanced analyses. For details of the file formats in which data are stored, see section S4.

```
%% load file name_Scan_Hopping
close all
clear;
Name='ScanHopping';%file name input

%% Load the file
loadname=strcat(Name,'.tsv');
A=load(loadname);
%% Set the columns for each data channel
chX = 1;% set data channel for X position
chY = 2;% set data channel for Y position
chZ = 3;% set data channel for Z position
chLN = 10;% set data channel for Line Number

premature_stop=0; %1==If the scan was stopped in the middle, 0==Complete scan

%% Set parameters from .set file
hopping_distance = 0.05;%HoppingDistance (µm)
x=round((max(A(chX,:))-min(A(chX,:)))/hopping_distance)+1;%no. of lines in x
direction
if premature_stop==1;
    y=round((max(A(chY,:))-min(A(chY,:)))/hopping_distance)-1;%no. of lines
in y direction
else
    y=round((max(A(chY,:))-min(A(chY,:)))/hopping_distance);%no. of lines in
y direction
end
first_approach=2;%Line Number of first approach
lines_in_hop=3;%Change in Line Number at every hop
last_line=first_approach+lines_in_hop*(x*y);

%% Generate point index of every hop
for i=first_approach:lines_in_hop:last_line
    b=A(:,A(chLN,:)==i);
```

```
    hop_no=(i-first_approach)/lines_in_hop+1;
    zdata=b(chZ,:);
    ZMap(hop_no)=zdata(end);%take the last data point of Z position at every
hop(e.g., approach)
end

%% Set X and Y range for the topography plot
XMIN=1;%pixel number you want to crop from and to
XMAX=x;
YMIN=1;
YMAX=y;
%% Plot Z Map (topography)
ZMapraw1 = ZMap;
ZMapraw1 = -(ZMapraw1-max(ZMapraw1)); % flip and zero the topographic data so
the higher features (shorter approaches) appear positive.
ZMapraw2 = ZMapraw1;
ZMapraw2 = ZMapraw2(1,1:x*y);
ZMapraw2 = reshape(ZMapraw2,x,y);
ZMapraw2 = ZMapraw2-(min(min(ZMapraw2)));% set the lowest position to 0
Toplot=ZMapraw2';
    for i=2:2:y
        Toplot(i,:)=fliplr(Toplot(i,:)); % flip every second line left-to-
right for account for serpentine scanning path
    end

Toplot=flipud(Toplot);
Toplot=Toplot(YMIN:YMAX,XMIN:XMAX);
figure;
imagesc(Toplot);

%Format plot
    title('Topography','fontsize',15);

cbar=colorbar('TickLength',[0],'LineWidth',1,'FontWeight','bold','FontSize',1
5);
    ylabel(cbar,['Surface Height / \mum'], 'Interpreter', 'tex', 'FontName',
'Arial','fontsize',15,'fontweight','bold','linewidth',1)
    axis normal
    axis equal
    axis tight
    set(gca,'xtick',[XMIN,XMAX],'ytick',[YMIN,YMAX])
    set(gca,'XTickLabel',[0,hopping_distance*(XMAX-
XMIN)],'YTickLabel',[0,hopping_distance*(YMAX-YMIN)])
    xlabel(['x / \mum'], 'Interpreter', 'tex','FontName', 'Arial')
    ylabel(['y / \mum'], 'Interpreter', 'tex', 'FontName', 'Arial')

    set(gca,'fontsize',15);
    set(gca,'TickDir','out');
    set(gca,'fontweight', 'bold');
    colormap gray
 %data saving
    topoFullname=strcat(Name,'-Z_Unprocessed');% save the topography
    file2Fullname=strcat(topoFullname,'.tsv');
    dlmwrite(file2Fullname,Toplot);% save the Toplot to .tsv for further data
processing
    saveas(gcf,topoFullname,'fig');% save the topography as a MATLAB figure
```

S7: Access to software and designs

The LabVIEW-based WEC-SPM code is freely available from a git repository; access can be obtained via completing a request on https://warwick.ac.uk/fac/sci/chemistry/research/unwin/electrochemistry/WEC-SPM/. Code and LabVIEW installation and configuration are described in detail in the associated configuration documentation that comes in the WEC-SPM code repository. Note, the GitHub repository allows code updates and improvements to be quickly and easily accessed by users. In addition, new custom experiment-specific user interfaces can be quickly and easily shared amongst the user community via upload to the GitHub repository.

In addition to the experimental code, the repository also contains open-source parts (see Supporting Information, section S2) and documentation, in the form of a user's guide, a developer's guide, and the academic-use open-source license agreement. The developer's guide is a 'live document' that is updated whenever new features are added, ensuring all options are appropriately documented.

S8: Additional experimental details for examples 1 and 2 from the main text

Instrumentation

A detailed description of the components used to build the SEPMs used in Examples 1 and 2 in the main text is provided in reference 34.

Experimental details

In both examples, nanopipettes were prepared from pulling quartz capillaries (1 mm o.d., 0.5 mm i.d., Friedrich & Dimmock) in a laser puller (P-2000, Sutter instruments; pulling parameters: (line 1) Heat 750, Fil 4, Vel 30, Del 150, and Pul 80; (line 2) Heat 650, Fil 3, Vel 40, Del 135, and Pul 150)). This resulted in nanopipettes with apertures that were 30-50 nm in diameter. Pipettes were back-filled with the solutions stated in the text.

Chloridized Ag wire (Example 1) or a hydrogen-saturated Pd wire electrode (see reference 36 for details of preparation; Example 2) were used quasi-reference/counter electrodes inside the pipette and in the bath solution.

S9: References

(1) Glasscott, M. W.; Brown, E. W.; Dorsey, K.; Laber, C. H.; Conley, K.; Ray, J. D.; Moores, L. C.; Netchaev, A. Selecting an Optimal Faraday Cage to Minimize Noise in Electrochemical Experiments. *Anal. Chem.* **2022**, *94* (35), 11983–11989. https://doi.org/10.1021/acs.analchem.2c02347.

(2) Kim, J.; Shen, M.; Nioradze, N.; Amemiya, S. Stabilizing Nanometer Scale Tip-to-Substrate Gaps in Scanning Electrochemical Microscopy Using an Isothermal Chamber for Thermal Drift Suppression. *Anal. Chem.* **2012**, *84* (8), 3489–3492. https://doi.org/10.1021/ac300564g.

(3) Zoski, C. G. Ultramicroelectrodes: Design, Fabrication, and Characterization. *Electroanalysis* **2002**, *14* (15–16), 1041–1051. https://doi.org/10.1002/1521-4109(200208)14:15/16<1041::AID-ELAN1041>3.0.CO;2-8.

(4) Cox, J. T.; Zhang, B. Nanoelectrodes: Recent Advances and New Directions. *Annu. Rev. Anal. Chem.* **2012**, *5* (1), 253–272. https://doi.org/10.1146/annurev-anchem-062011-143124.

(5) Polcari, D.; Dauphin-Ducharme, P.; Mauzeroll, J. Scanning Electrochemical Microscopy: A Comprehensive Review of Experimental Parameters from 1989 to 2015. *Chem. Rev.* **2016**, *116* (22), 13234–13278. https://doi.org/10.1021/acs.chemrev.6b00067.

(6) Perry, D.; Momotenko, D.; Lazenby, R. A.; Kang, M.; Unwin, P. R. Characterization of Nanopipettes. *Anal. Chem.* **2016**, *88* (10), 5523–5530. https://doi.org/10.1021/acs.analchem.6b01095.

(7) *Xometry*. https://www.xometry.com/ (accessed 2025-08-17).

(8) *Hubs*. https://www.hubs.com (accessed 2025-08-17).

(9) *Protolabs*. https://www.protolabs.com/ (accessed 2025-08-17).

(10) Ebejer, N.; Schnippering, M.; Colburn, A. W.; Edwards, M. A.; Unwin, P. R. Localized High Resolution Electrochemistry and Multifunctional Imaging: Scanning Electrochemical Cell Microscopy. *Anal. Chem.* **2010**, *82* (22), 9141–9145. https://doi.org/10.1021/ac102191u.

(11) Wipf, D. O.; Bard, A. J. Scanning Electrochemical Microscopy. 15. Improvements in Imaging via Tip-Position Modulation and Lock-in Detection. *Anal. Chem.* **1992**, *64* (13), 1362–1367. https://doi.org/10.1021/ac00037a011.

(12) Shevchuk, A. I.; Gorelik, J.; Harding, S. E.; Lab, M. J.; Klenerman, D.; Korchev, Y. E. Simultaneous Measurement of Ca^{2+} and Cellular Dynamics: Combined Scanning Ion Conductance and Optical Microscopy to Study Contracting Cardiac Myocytes. *Biophys. J.* **2001**, *81* (3), 1759–1764. https://doi.org/10.1016/S0006-3495(01)75826-2.

(13) *The NI TDMS File Format*. https://www.ni.com/en-au/support/documentation/supplemental/06/the-ni-tdms-file-format.html (accessed 2025-08-14).

(14) *tdmsread documentation*. https://www.mathworks.com/help/daq/tdmsread.html (accessed 2025-08-17).

(15) *TDMS excel add-in*. https://www.ni.com/en/support/downloads/tools-network/download.tdm-excel-add-in-for-microsoft-excel.html (accessed 2025-08-17).

(16) *npTDMS package*. https://pypi.org/project/npTDMS/ (accessed 2025-08-17).

(17) Aaronson, B. D. B.; Byers, J. C.; Colburn, A. W.; McKelvey, K.; Unwin, P. R. Scanning Electrochemical Cell Microscopy Platform for Ultrasensitive Photoelectrochemical Imaging. *Anal. Chem.* **2015**, *87* (8), 4129–4133. https://doi.org/10.1021/acs.analchem.5b00288.

(18) Chen, C.-H.; Jacobse, L.; McKelvey, K.; Lai, S. C. S.; Koper, M. T. M.; Unwin, P. R. Voltammetric Scanning Electrochemical Cell Microscopy: Dynamic Imaging of Hydrazine Electro-Oxidation on Platinum Electrodes. *Anal. Chem.* **2015**, *87* (11), 5782–5789. https://doi.org/10.1021/acs.analchem.5b00988.

(19) McKelvey, K.; Kinnear, S. L.; Perry, D.; Momotenko, D.; Unwin, P. R. Surface Charge Mapping with a Nanopipette. *J. Am. Chem. Soc.* **2014**, *136* (39), 13735–13744. https://doi.org/10.1021/ja506139u.

(20) McKelvey, K.; Perry, D.; Byers, J. C.; Colburn, A. W.; Unwin, P. R. Bias Modulated Scanning Ion Conductance Microscopy. *Anal. Chem.* **2014**, *86* (7), 3639–3646. https://doi.org/10.1021/ac5003118.

(21) Paulose Nadappuram, B.; McKelvey, K.; Al Botros, R.; Colburn, A. W.; Unwin, P. R. Fabrication and Characterization of Dual Function Nanoscale pH-Scanning Ion Conductance Microscopy (SICM) Probes for High Resolution pH Mapping. *Anal. Chem.* **2013**, *85* (17), 8070–8074. https://doi.org/10.1021/ac401883n.

(22) Paulose Nadappuram, B.; McKelvey, K.; Byers, J. C.; Güell, A. G.; Colburn, A. W.; Lazenby, R. A.; Unwin, P. R. Quad-Barrel Multifunctional Electrochemical and Ion Conductance Probe for Voltammetric Analysis and Imaging. *Anal. Chem.* **2015**, *87* (7), 3566–3573. https://doi.org/10.1021/acs.analchem.5b00379.

(23) Patel, A. N.; Collignon, M. G.; O'Connell, M. A.; Hung, W. O. Y.; McKelvey, K.; Macpherson, J. V.; Unwin, P. R. A New View of Electrochemistry at Highly Oriented Pyrolytic Graphite. *J. Am. Chem. Soc.* **2012**, *134* (49), 20117–20130. https://doi.org/10.1021/ja308615h.

(24) Ebejer, N.; Schnippering, M.; Colburn, A. W.; Edwards, M. A.; Unwin, P. R. Localized High Resolution Electrochemistry and Multifunctional Imaging: Scanning

Electrochemical Cell Microscopy. *Anal. Chem.* **2010**, *82* (22), 9141–9145. https://doi.org/10.1021/ac102191u.

(25) Momotenko, D.; Byers, J. C.; McKelvey, K.; Kang, M.; Unwin, P. R. High-Speed Electrochemical Imaging. *ACS Nano* **2015**, *9* (9), 8942–8952. https://doi.org/10.1021/acsnano.5b02792.

(26) Momotenko, D.; McKelvey, K.; Kang, M.; Meloni, G. N.; Unwin, P. R. Simultaneous Interfacial Reactivity and Topography Mapping with Scanning Ion Conductance Microscopy. *Anal. Chem.* **2016**, *88*, 2838–2846. https://doi.org/10.1021/acs.analchem.5b04566.

(27) Page, A.; Perry, D.; Young, P.; Mitchell, D.; Frenguelli, B. G.; Unwin, P. R. Fast Nanoscale Surface Charge Mapping with Pulsed-Potential Scanning Ion Conductance Microscopy. *Anal. Chem.* **2016**, *88* (22), 10854–10859. https://doi.org/10.1021/acs.analchem.6b03744.

(28) Bentley, C. L.; Andronescu, C.; Smialkowski, M.; Kang, M.; Tarnev, T.; Marler, B.; Unwin, P. R.; Apfel, U-P.; Schuhmann, W. Local Surface Structure and Composition Control the Hydrogen Evolution Reaction on Iron Nickel Sulfides. *Angew. Chem. Int. Ed.* **2018**, *57* (15), 4093–4097. https://doi.org/10.1002/anie.201712679.

(29) Bentley, C. L.; Kang, M.; Maddar, F. M.; Li, F.; Walker, M.; Zhang, J.; Unwin, P. R. Electrochemical Maps and Movies of the Hydrogen Evolution Reaction on Natural Crystals of Molybdenite (MoS_2): Basal *vs.* Edge Plane Activity. *Chem. Sci.* **2017**, *8* (9), 6583–6593. https://doi.org/10.1039/c7sc02545a.

(30) Bentley, C. L.; Kang, M.; Unwin, P. R. Nanoscale Structure Dynamics within Electrocatalytic Materials. *J. Am. Chem. Soc.* **2017**, *139* (46), 16813–16821. https://doi.org/10.1021/jacs.7b09355.

(31) Page, A.; Kang, M.; Armitstead, A.; Perry, D.; Unwin, P. R. Quantitative Visualization of Molecular Delivery and Uptake at Living Cells with Self-Referencing Scanning Ion Conductance Microscopy-Scanning Electrochemical Microscopy. *Anal. Chem.* **2017**, *89* (5), 3021–3028. https://doi.org/10.1021/acs.analchem.6b04629.

(32) Zhang, G.; Güell, A. G.; Kirkman, P. M.; Lazenby, R. A.; Miller, T. S.; Unwin, P. R. Versatile Polymer-Free Graphene Transfer Method and Applications. *ACS App. Mater. Interfaces* **2016**, *8* (12), 8008–8016. https://doi.org/10.1021/acsami.6b00681.

(33) Perry, D.; Page, A.; Chen, B.; Frenguelli, B. G.; Unwin, P. R. Differential-Concentration Scanning Ion Conductance Microscopy. *Anal. Chem.* **2017**, *89* (22), 12458–12465. https://doi.org/10.1021/acs.analchem.7b03543.

(34) Kang, M.; Perry, D.; Bentley, C. L.; West, G.; Page, A.; Unwin, P. R. Simultaneous Topography and Reaction Flux Mapping at and around Electrocatalytic Nanoparticles. *ACS Nano* **2017**, *11* (9), 9525–9535. https://doi.org/10.1021/acsnano.7b05435.

(35) Chen, B.; Perry, D.; Page, A.; Kang, M.; Unwin, P. R. Scanning Ion Conductance Microscopy: Quantitative Nanopipette Delivery–Substrate Electrode Collection Measurements and Mapping. *Anal. Chem*. **2019**, *91* (3), 2516–2524. https://doi.org/10.1021/acs.analchem.8b05449.

(36) Ustarroz, J.; Ornelas, I. M.; Zhang, G.; Perry, D.; Kang, M.; Bentley, C. L.; Walker, M.; Unwin, P. R. Mobility and Poisoning of Mass-Selected Platinum Nanoclusters during the Oxygen Reduction Reaction. *ACS Catal*. **2018**, *8* (8), 6775–6790. https://doi.org/10.1021/acscatal.8b00553.
